# A sugar utilization phenotype contributes to the formation of genetic exchange communities in lactic acid bacteria

**DOI:** 10.1093/femsle/fnab117

**Published:** 2021-09-01

**Authors:** Shinkuro Takenaka, Takeshi Kawashima, Masanori Arita

**Affiliations:** Department of Genetics, The Graduate University for Advanced Studies, SOKENDAI, Mishima, Shizuoka 411-8540, Japan; Department of Genetics, The Graduate University for Advanced Studies, SOKENDAI, Mishima, Shizuoka 411-8540, Japan; National Institute of Genetics, Mishima, Shizuoka 411-8540, Japan; Department of Genetics, The Graduate University for Advanced Studies, SOKENDAI, Mishima, Shizuoka 411-8540, Japan; National Institute of Genetics, Mishima, Shizuoka 411-8540, Japan

**Keywords:** lactic acid bacteria, accessory genes, distribution, ortholog analysis, genetic exchange community, ecological niche

## Abstract

In prokaryotes, a major contributor to genomic evolution is the exchange of genes via horizontal gene transfer (HGT). Areas with a high density of HGT networks are defined as genetic exchange communities (GECs). Although some phenotypes associated with specific ecological niches are linked to GECs, little is known about the phenotypic influences on HGT in bacterial groups within a taxonomic family. Thanks to the published genome sequences and phenotype data of lactic acid bacteria (LAB), it is now possible to obtain more detailed information about the phenotypes that affect GECs. Here, we have investigated the relationship between HGT and internal and external environmental factors for 178 strains from 24 genera in the *Lactobacillaceae* family. We found a significant correlation between strains with high utilization of sugars and HGT bias. The result suggests that the phenotype of the utilization of a variety of sugars is key to the construction of GECs in this family. This feature is consistent with the fact that the *Lactobacillaceae* family contributes to the production of a wide variety of fermented foods by sharing niches such as those in vegetables, dairy products and brewing-related environments. This result provides the first evidence that phenotypes associated with ecological niches contribute to form GECs in the LAB family.

## Teaser

The ability to utilize a variety of sugars contributed to increased horizontal gene transfer and the formation of genetic exchange communities in the ecological niches among lactic acid bacteria.

## INTRODUCTION

Horizontal gene transfer (HGT) is an evolutionary process that allows for the spread of genetic innovations between distantly related organisms (Andam and Gogarten 2011). Among microorganisms, HGT plays a major role in the rapid sharing of biological features (Andam and Gogarten 2011) and can result in large changes to the genome size (Zimmer and Emlen 2016). Variability in the genome size is also frequently observed among closely related strains (Canard and Cole 1989; Daniels 1990; Tanskanen *et al*. 1990; Prevost *et al*. 1992; Harsono *et al*. 1993), and this can also be caused by HGT (Bergthorsson and Ochman 1995; Bobay and Ochman 2017). When such transfer is described as networks (Puigbò *et al*. 2010), the HGT bias by preferences for transfer partners results in high-density regions in the networks, defined as genetic exchange communities (GECs; Skippington and Ragan 2011). GECs often occur in shared ecological niches, characterized by symbiotic interactions and phylogenetic closeness (Andam and Gogarten 2011).

GECs in shared ecological niches influence microbial evolution, as they provide a selective advantage to microbes, which then allows for their expansion into new ecological niches (Swithers *et al*. 2012; Soucy *et al*. 2015). However, this complicates the evolution or adaptation within the same GECs (Polz *et al*. 2013). Ragan and Beiko (2009) suggested that the habitats of donors and recipients are key limitations for HGT, we need to further investigate the impacts of how environmental range constrains HGT, as it may have thus far been underestimated.

To better understand the influence of ecological niches on HGT, the relationship of the microorganism's phenotypes to environmental adaptation should be investigated. Phenotypes such as those for resource utilization enable microbes to survive in a variety of environments and thus help define the range of the microbes' habitat (Chen *et al*. 2021). Jain *et al*. (2003) investigated the internal and external environmental factors that regulate HGT in eight bacterial and archaeal genomes. They reported that HGT occurs among organisms that share similar factors including host phenotype, such as carbon utilization and oxygen tolerance. Their analyses provided evidence for the effects of GECs in ecological niches on prokaryote evolution. However, it is unclear if this tendency is applicable to GECs formed by bacterial groups of same family in particular ecosystem niches. This is because the HGT among related bacterial groups is affected not only by the bias of the ecological niche they share but also the bias of their closely related partners with whom they preferentially exchange genes (Andam and Gogarten 2011; Soucy *et al*. 2015). To clarify this point in more detail, a comparative analysis using a large amount of phenotypic and genomic data for related species is required.

Here, we have investigated the genomic and phenotypic features for 178 strains of 24 genera from the *Lactobacillaceae* family to clarify factors contributing to the formation of GECs. Lactic acid bacteria (LAB) produce lactic acid by fermenting carbohydrates and inhabit specific ecological niches, such as fermented milk products, meats, cereals and vegetables (Caplice and Fitzgerald 1999). The genus *Lactobacillus* has recently been reclassified into 25 genera by Zheng *et al*. (2020), and provides an adequate sandbox to study the influence of ecological niches on HGT in relation with phenotypes, ecologies and genotypes. Their phenotypes such as sugar utilization, growth temperature and oxygen tolerance have been well investigated and documented (Holzapfel and Wood 2014).

## MATERIALS AND METHODS

### Genome sequences of *Lactobacillaceae* family and their features

The genome sequences and genomic features of 178 strains, previously identified as the genus *Lactobacillus*, were retrieved from the DFAST Archive of Genome Annotation (https://dfast.nig.ac.jp/genomes/; Tanizawa *et al*. 2016) database. A total of six genomic features (genome size, number of coding sequences (CDS), GC content, number of genes encoding rRNAs, number of genes encoding tRNAs and number of CRISPRs) were used in this study.

The sequences for the 16S rRNA genes were obtained from EZBioCloud (https://www.ezbiocloud.net/resources/16s_download). A total of six phenotypic data of these strains (the number of sugars they can metabolize (sugar utilization value), growth at 15°C, growth at 45°C, microaerobic growth, facultatively anaerobic growth and obligate anaerobic growth) were obtained from the book ‘Lactic Acid Bacteria: Biodiversity and Taxonomy (Holzapfel and Wood 2014).’ Isolation sources for the *Lactobacillaceae* family were obtained from the paper of Zheng *et al*. (2020). Table S1 (Supporting Information) shows the correspondence between old and new species names, genomic features, phenotypic features and isolation sources.

### HGT analysis

Genes acquired via HGT were predicted by the DarkHorse v2.0 (Podell and Gaasterland 2007) and COLOMBO v4.0 analysis with SIGI-HMM (Waack *et al*. 2006). DarkHorse and COLOMBO were run with default parameters. The CDSs were judged as HGT when their lineage probability index was ≥ 0.5 (DarkHorse) or annotation was PUTAL (COLOMBO).

### Construction of the *Lactobacillaceae* family phylogenetic tree

Phylogenetic trees for the 178 strains was constructed based on the 16S rRNA gene and the genes clustered by ortholog analysis. To generate the phylogenetic tree, MUSCLE, Multiple Sequence Alignment (Edgar 2004) and the neighbor joining method (Saitou and Nei 1987) were implemented and performed using the program MEGA (Kumar *et al*. 2018). The 16S rRNA tree was annotated using iTOL (Letunic and Bork 2007).

### Multiple regression analysis between size of genome or number of HGT genes and *Lactobacillaceae* family features

Multiple regression analysis was performed using the python package Statsmodels (https://www.statsmodels.org/stable/). Dummy variables (1 for yes and 0 for no) were used for the following five features: growth at 15°C, growth at 45°C and growth in microaerobic, facultatively anaerobic and obligate anaerobic conditions. For the strains with missing phenotypic data, average values from all the other strains were assigned. All explanatory variables were normalized using a Z score transformation.

### Ortholog analysis

Orthologs for 178 strains of *Lactobacillaceae* family were obtained using SonicParanoid software (Cosentino and Iwasaki 2019) with the default parameters. Strain-specific genes were discarded.

### Core- and accessory-genome computation and COG assignment

For core- and accessory-genome analysis, we used clusters of orthologous group (COG) functional categories to classify the functions of the gene clusters for the 178 genomes of *Lactobacillaceae* family (http://www.ncbi.nlm.nih.gov/COG/). Using ortholog analysis data with COG annotation, we determined the core- and accessory-genomes based on the method described by Satti *et al*. (2018). The method selects an appropriate *n*-core which is the set of genes conserved in *n*% of the genomes based on the COG information for the orthologs. We created 10 *n*-cores from 100- to 91-core, and finally the 97-core was selected as the core-genome for this analysis.

### Calculation of average of sugar utilization for the orthologs

To estimate the characteristics for each ortholog, we calculated the average number of metabolizable sugars of strains for each ortholog cluster as the Average number of Sugar Utilization for the ortholog (ASU). Statistically meaningful orthologs were chosen based on their ASU as their standard deviation is more/less than 1 from the average of sugar utilization value in the 178 strains. The COG number for the chosen orthologs were counted and the ratio of each group was statistically analyzed using a *t*-test and Benjamini–Hochberg correction for multiple comparisons, using the Python package Statsmodels (https://www.statsmodels.org/stable/).

### Construct networks of sharing ortholog

A network graph was constructed for the selected orthologs using ASU value. Each of the 178 nodes represents a genome of *Lactobacillaceae* family and an edge was created between two genomes when the number of shared orthologs was more than five. Community extraction and visualization were performed with the Python package NetworkX (https://networkx.org/) and with CytoScape (version 3.8.2; Smoot *et al*. 2011), respectively.

## RESULTS

### Relationship among the phylogenetic, genomic and phenotypic features in 178 strains from the *Lactobacillaceae* family

**Figure 1.**
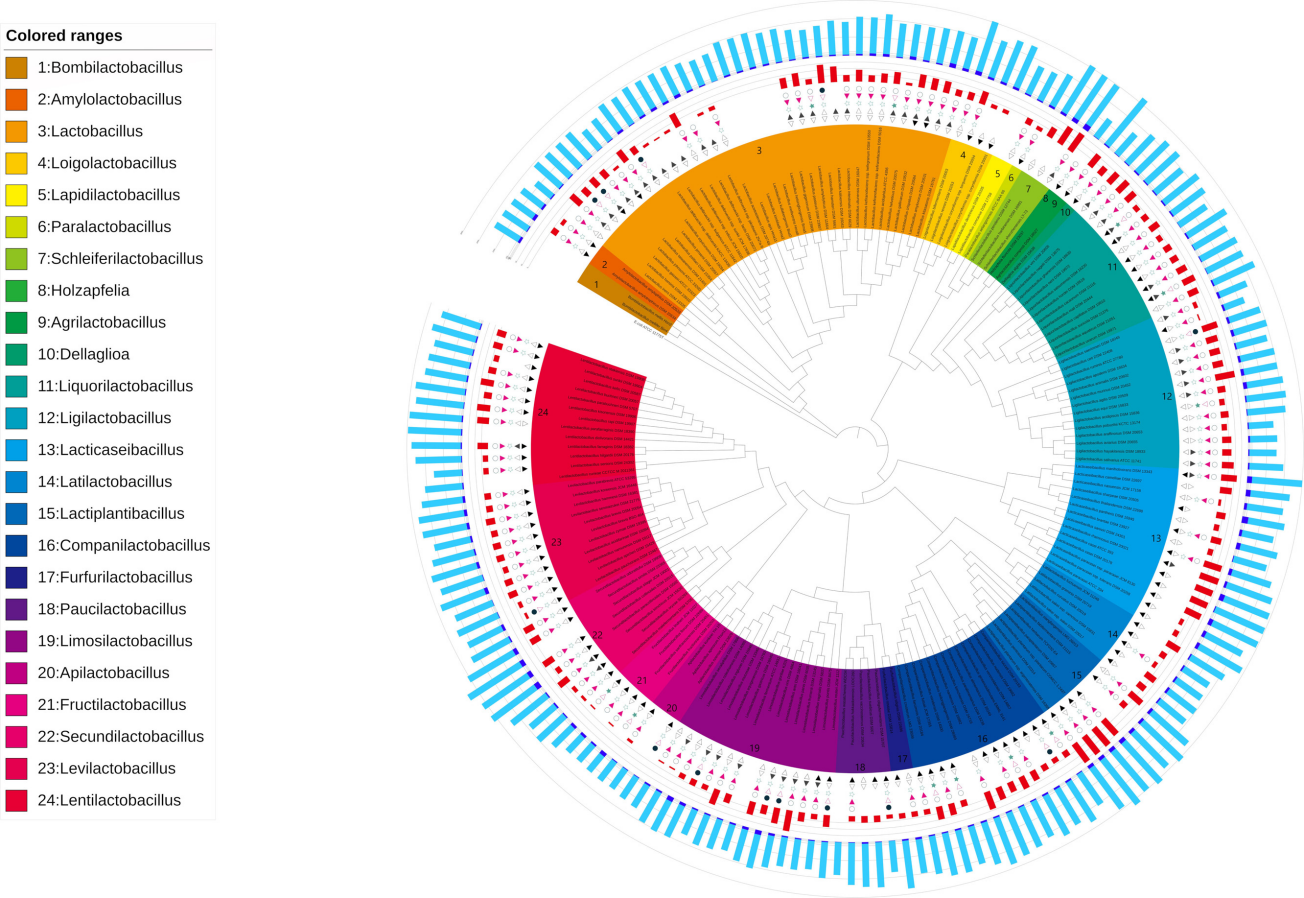
Phylogenetic tree based on the 16S rRNA genes of the LAB strains with the phenotypic and genomic features identified. The inner band shows species colored by genus. The next five symbols show phenotypic characteristics for each LAB strain; first inward-facing triangle indicates the growth at 15°C, second outward-facing triangle indicates the growth at 45°C, third star indicates the micro aerophilic, fourth red inward-facing indicates facultatively anaerobic and fifth circle indicates obligate anaerobic. A filled symbol means the strain has the phenotype, and an open symbol means that it does not. A blank means that there is no relevant information available. The next red band shows the number of sugar types that can be utilized. The outer bands show the number of coding sequences (CDS) for each strain: navy blue indicates the estimated number of CDS acquired by the horizontal gene transfer (HGT) and light blue indicates the number of native CDS.

We first examined the phenotypic and genomic features of each of the 178 strains and mapped them onto a phylogenetic tree (Fig. 1). A total of six phenotypes were assessed: two conditions for temperature required for bacterial growth (ability to grow at 15 and 45°C), three conditions for oxygen tolerance (microaerobic, facultatively anaerobic and obligate anaerobic) and the sugar utilization value (the number of sugars each strain can metabolize). Of the 178 strains, 56.8% grew at 15°C and 33.3% grew at 45°C. Among these strains, 8.3%, 81.9% and 9.8% were microaerobic, facultatively anaerobic and obligate anaerobic, respectively. Sugar utilization values ranged from 0 to 17 (excluding glucose), and the average for all strains was 6.83. For the genomic feature, we investigated the number of total CDS for each strain and estimated the number of CDS gained via HGT. The total number of CDS for each of the 178 strains ranged from 1191 to 3600. Since the total number of CDS and the genome size were strongly correlated (*R* = 0.976), they were treated as interchangeable information in this analysis. The number of CDS gained via HGT ranged from 17 to 342 (Table S1, Supporting Information), and indicated a weak correlation with genome size (*R* = 0.394) and the total number of CDS (*R* = 0.424).

Variation was observed in the phenotypic features of the groups clustered by the phylogenetic tree (Fig. 1). In particular, the sugar utilization values varied even within the same genus. For example, in the group for the genus *Lactobacillus*, although *Lactobacillus iners* had the lowest sugar-type utilization profile of 0, *Lactobacillus hamster* could utilize 14 kinds of sugar. In addition, the sugar utilization values of the *Ligilactobacillus* genus ranged from 1 to 15 and that of the *Limosilactobacillus* genus ranged from 1 to 16.

The correspondence between the numbers of CDS in a genome and the sugar utilization values were observed (Fig. 1). The tendency was remarkable in the clusters for the genera *Ligilactobacillus*, *Lacticaseibacillus*, *Limosilactobacillus*, *Apilactobacillus*, *Fructilactobacillus* and *Secundilactobacillus*. For example, *Lacticaseibacillus manihotivorans*, *Lacticaseibacillus saniviri*, *Lacticaseibacillus casei* and *Lacticaseibacillus paracasei* ssp. *paracasei* had high numbers of CDS and high sugar utilization values, while *Lacticaseibacillus nasuensis*, *Lacticaseibacillus thailandensis* and *Lacticaseibacillus brantae* had low numbers of CDS and low sugar utilization values.

### Influence of phenotypic features on genome size and number of HGT genes

To confirm the relationship between genomic features and sugar utilization suggested in Fig. 1, the following multiple regression analyses were performed. The six phenotypic (sugar utilization value, growth at 15°C, growth at 45°C and growth in microaerobic, facultatively anaerobic and obligate anaerobic conditions) and four genomic features (G/C content, number of rRNA genes, number of tRNA genes and number of CRISPRs) were subjected to multiple regression analysis as explanatory variables (Table S1, Supporting Information).

**Figure 2.**
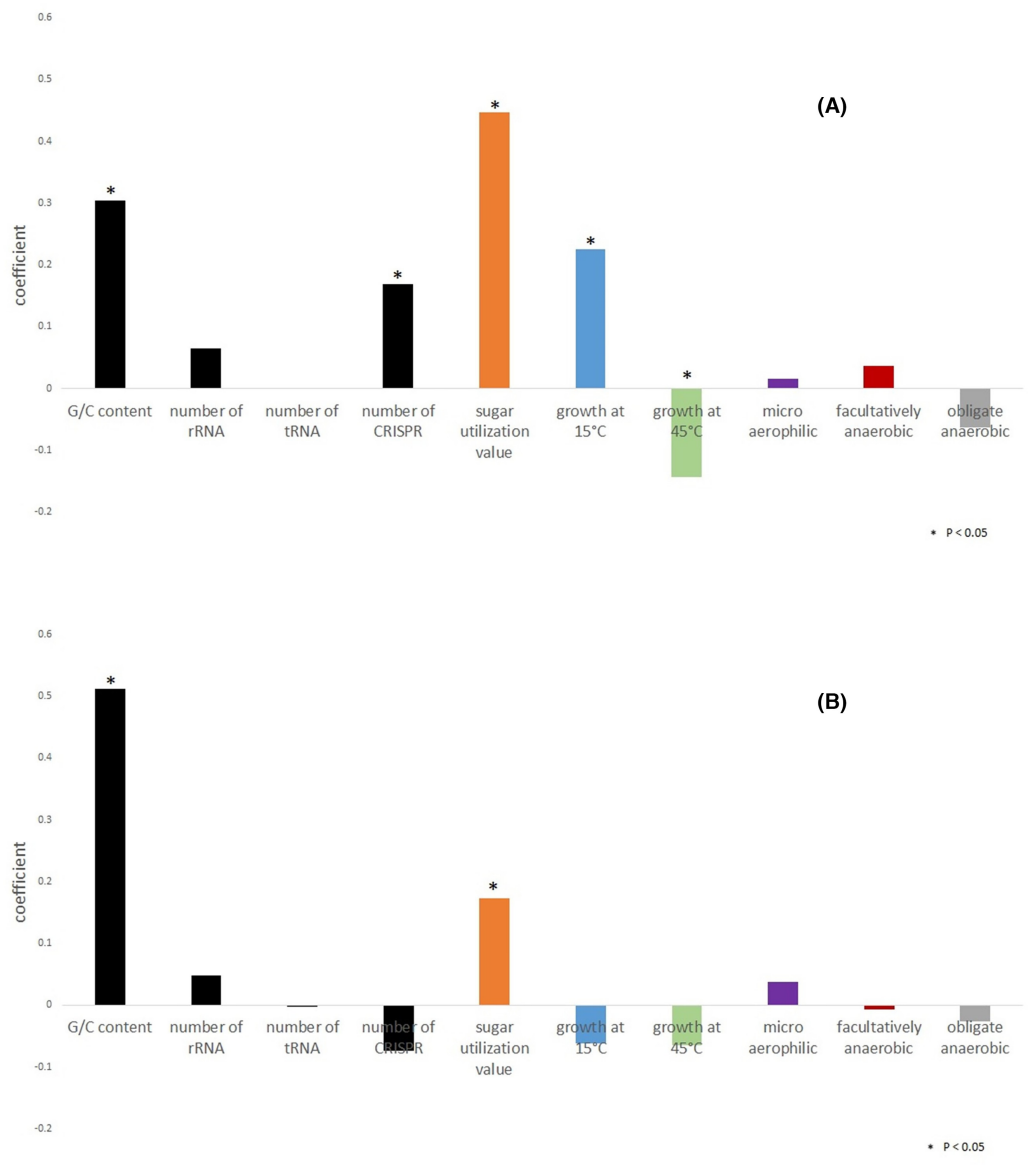
Values of the coefficients of the multiple aggression analysis for **(A)** genome size and **(B)** the number of CDS judged to be HGTs. The genome size or number of CDS judged to be HGTs was set as the objective variable, and the six phenotypic features (sugar utilization value, growth at 15°C, growth at 45°C, microaerobic, facultatively anaerobic and obligate anaerobic) and four genomic features (G/C content, number of rRNAs, number of tRNAs and number of CRISPRs) were subjected to multiple regression analysis as explanatory variables. * indicates a *P*-value ≤ 0.05.

The genome sizes of 178 strains in *Lactobacillaceae* family were set as the objective variable. The six phenotypic features and the four genomic features were set as the explanatory variables. The coefficient of determination (R2) obtained was 0.484, and the correlation coefficient (R) was 0.696. For sugar utilization values, growth at 15°C, growth at 45°C, G/C content and number of CRISPRs, *P-*value was < 0.05. The coefficient of growth at 45°C was negative and that of G/C content, growth at 15°C and number of CRISPRs were positive. The sugar utilization value had the largest coefficient among these factors (Fig. 2A).

CDS that were transferred from other taxa (HGT gene) were also set as an objective variable and the ten factors used to analyze the genome size were set as explanatory variables. As a result, the coefficient of determination (R2) obtained was 0.298, and the correlation coefficient (R) was 0.546. For both the sugar utilization value and the G/C composition, *P* value was < 0.05 and they had a positive correlation (Fig. 2B).

### COG ratios of orthologs in the core- and accessory-genome

Hereafter, we detected HGT among strains in *Lactobacillaceae* family by the combination of ortholog and network analyses (see Methods), because the above methods (DarkHorse and COLOMBO software) are suitable only for detecting HGTs between distantly related organisms.

To understand the characteristics of HGT genes in *Lactobacillaceae* family, we focused on "accessory genomes’. The variable portion of the genome that is present between individual strains is often called the ‘accessory genome’ and differs from the core genome (Sim *et al*. 2008). Here, we compared the functions in accessory genomes except strain-specific singletons to the functions in core genomes.

To classify all genes into core and accessory genomes, we first conducted an ortholog analysis for the CDS present in the 178 strains and found that the 384 737 putative protein sequences were grouped into 12 884 ortholog clusters. The core- and accessory-genomes were determined using the COG assignment of each ortholog. The number of core-genes and accessory genes corresponded to 532 and 12 352 ortholog clusters, respectively. The COG ratios of the core- and accessory-genomes were quite different (Fig. 4). Metabolism related genes were enriched in the accessory genomes.

### Ortholog features shared by generalists or specialists for sugar utilization

**Figure 3.**
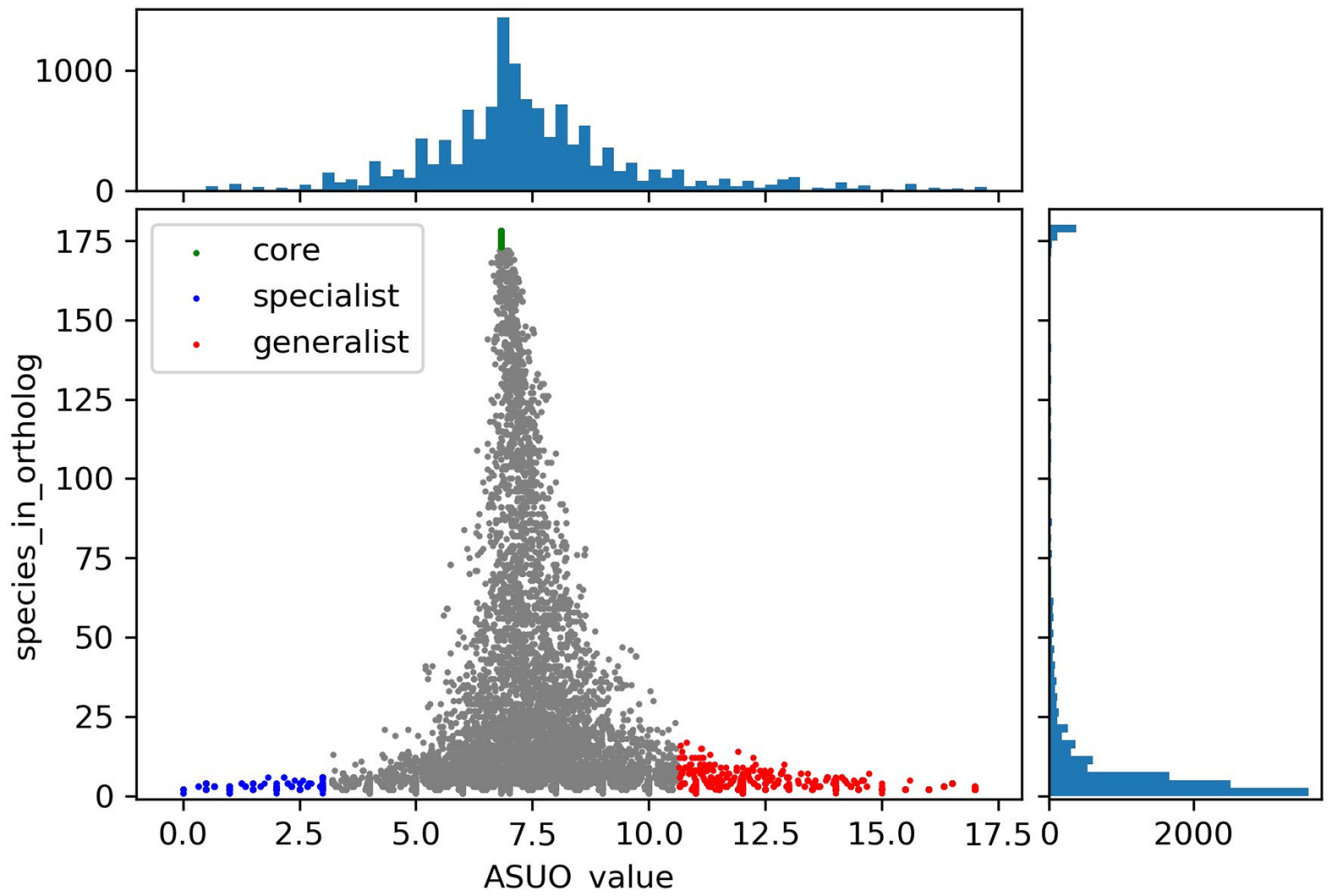
ASU value and number of strains for each ortholog. The vertical axis indicates the number of strains in each ortholog, and the horizontal axis indicates the ASU value for each ortholog. We introduced the concept of ASU (Average of Sugar Utilization for the Ortholog) value. For example, two sequences derived from strains A and B that were clustered as an ortholog, then their ASU value was calculated as the average sugar utilization value for A and B. We also calculated the overall average and standard deviation of the sugar utilization value in 178 strains, then ortholog clusters were chosen when their ASU values were more/less than the means ± one standard deviation. The orthologs with high ASU values are designated as generalist group orthologs (red dots) and the low group are designated as specialist group orthologs (blue dots). Core genes from the 178 LAB strains are indicated as green dots. The top and side histograms show the number of orthologs on each axis.

To confirm that sugar utilization values influence HGT bias, the functions of two groups of orthologs were compared, i.e. the orthologs shared dominantly by strains which were able to use a variety of sugars (generalist) and those that use only few sugars (specialist). Here, we introduce the concept of the ASU value to extract generalist and specialist group orthologs (see material and method). The overall average and standard deviation of the sugar utilization values in all 178 strains were calculated. The ortholog clusters were selected when they had ASU values that were more or/less than the mean ± one standard deviations and they were designated as generalist/specialist group orthologs (Fig. 3). The generalist group orthologs tended to be shared by more strains.

**Figure 4.**
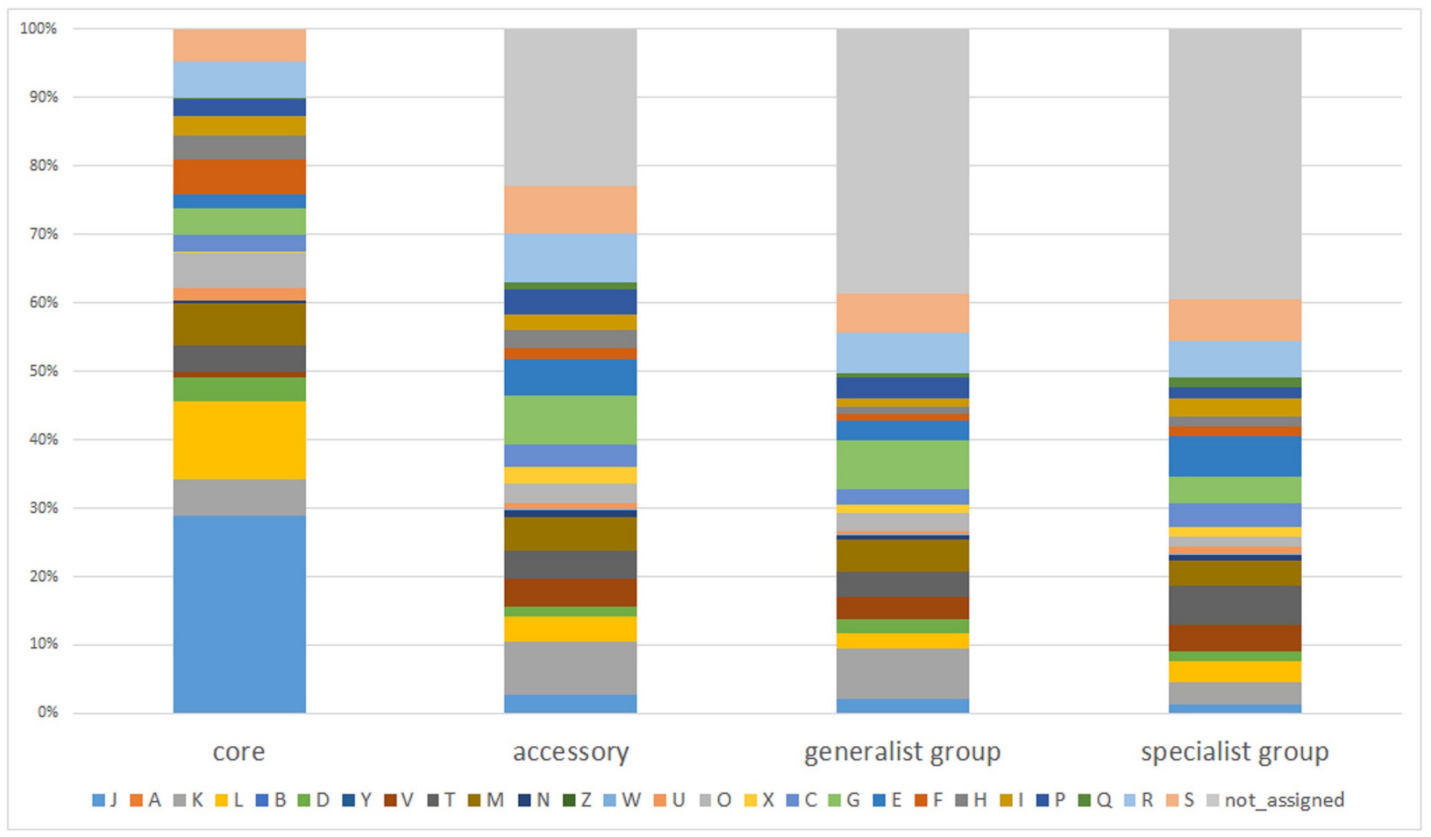
The clusters of orthologous group (COG) ratios for each group of orthologs. The COG ratios of the core genome, accessory genome, generalist group orthologs and specialist group orthologs are displayed. [J] Translation, ribosomal structure and biogenesis, [A] RNA processing and modification, [K] Transcription, [L] Replication, recombination and repair, [B] Chromatin structure and dynamics, [D] Cell cycle control, cell division and chromosome partitioning, [Y] Nuclear structure, [V] Defense mechanisms, [T] Signal transduction mechanisms, [M] Cell wall/membrane/envelope biogenesis, [N] Cell motility, [Z] Cytoskeleton, [W] Extracellular structures, [U] Intracellular trafficking, secretion and vesicular transport, [O] Post-translational modification, protein turnover and chaperones, [X] Mobilome: prophages and transposons, [C] Energy production and conversion, [G] Carbohydrate transport and metabolism, [E] Amino acid transport and metabolism, [F] Nucleotide transport and metabolism, [H] Coenzyme transport and metabolism, [I] Lipid transport and metabolism, [P] Inorganic ion transport and metabolism, [Q] Secondary metabolites biosynthesis, transport and catabolism, [R] General function prediction only and [S] Function unknown. Orthologs not assigned COG are indicated in gray color. In the accessory genome, more metabolism-related genes such as ‘carbohydrate transport and metabolism’ (G), ‘amino acid transport and metabolism’ (E), ‘transcription’ (K) and ‘defense mechanisms’ (V) were enriched than in the core genome. On the other hand, ‘translation, ribosomal structure and biogenesis’ (J) and ‘replication, recombination and repair’ (L) were lower than in the core genome.

The ratio of the COG functions between the generalist and specialist group orthologs showed no significant differences (Fig. 4 and Table 1). The result suggests that the genes are acquired neutrally in HGT, regardless of the phenotypic difference between generalist and specialist.

**Table 1.** *T*-test and Benjamini–Hochberg method to compare the functional ratio of COG for each group. The right side of the table indicates the *P*-value for the *t*-test to compare each COG ratio between all combinations to choose two from three groups (accessory genome, generalist group orthologs and specialist group orthologs). The left side of the table indicates the Boolean values of the Benjamini–Hochberg correction at a 0.05 false discovery rate (FDR) level. Significant differences indicate TRUE.

	*P*-value	*t*-test and Benjamini–Hochberg method				
COG	All accessory vs generalist	All accessory vs specialist	Generalist vs specialist	All accessory vs generalist	All accessory vs specialist	Generalist vs specialist
J	0.326 101	0.114 384	0.32 189	FALSE	FALSE	FALSE
A	0.770 197	0.86 256	ND	FALSE	FALSE	FALSE
K	0.660 644	0.001 324	0.005 024	FALSE	TRUE	FALSE
L	0.016 087	0.454 098	0.458 151	FALSE	FALSE	FALSE
B	ND	ND	ND	FALSE	FALSE	FALSE
D	0.233 915	0.902 782	0.498 252	FALSE	FALSE	FALSE
Y	ND	ND	ND	FALSE	FALSE	FALSE
V	0.253 986	0.908 512	0.590 247	FALSE	FALSE	FALSE
T	0.546 536	0.086 224	0.073 969	FALSE	FALSE	FALSE
M	0.609 181	0.285 109	0.484 595	FALSE	FALSE	FALSE
N	0.330 625	0.666 394	0.873 454	FALSE	FALSE	FALSE
Z	ND	ND	ND	FALSE	FALSE	FALSE
W	0.795 567	0.973 348	0.906 121	FALSE	FALSE	FALSE
U	0.164 648	0.519 524	0.133 258	FALSE	FALSE	FALSE
O	0.74 121	0.073 661	0.129 009	FALSE	FALSE	FALSE
X	0.003 727	0.155 424	0.688 248	FALSE	FALSE	FALSE
C	0.115 125	0.690 668	0.197 208	FALSE	FALSE	FALSE
G	0.971 753	0.014 538	0.025 503	FALSE	FALSE	FALSE
E	0.000 799	0.679 508	0.012 048	TRUE	FALSE	FALSE
F	0.062 515	0.913 128	0.279 673	FALSE	FALSE	FALSE
H	0.002 552	0.136 954	0.679 383	TRUE	FALSE	FALSE
I	0.018 139	0.633 887	0.046 447	FALSE	FALSE	FALSE
P	0.275 201	0.034 176	0.140 896	FALSE	FALSE	FALSE
Q	0.159 491	0.383 424	0.094 268	FALSE	FALSE	FALSE
R	0.149 804	0.147 752	0.581 598	FALSE	FALSE	FALSE
S	0.145 587	0.624 075	0.713 207	FALSE	FALSE	FALSE
Not_assigned	0	0	0.804 117	TRUE	TRUE	FALSE

Among the orthologs shared by the generalists for sugar utilization, some genes were found to be involved in adaptations to various niches (Table S3, Supporting Information). Some examples are as follows. Cell division protein FtsK (Diez *et al*. 2000), xenobiotic response element (XRE) family transcriptional regulator (Hu *et al*. 2018), and phenolic acid-responsive transcriptional regulator (PadR) family (Gury *et al*. 2004) are related to stress responses. Bacteriocin precursor peptides PlnE and PlnF (Anderssen *et al*. 1998) are related to bacteriocin production. The multiple antibiotic resistance protein (MarR) family transcriptional regulator (Silva *et al*. 2018) is related to antibiotic resistance. Peptide methionine sulfoxide reductase (Walter *et al*. 2005) is related to survival in the intestinal environment. Mercuric resistance operon regulatory protein (MerR) family transcriptional regulator (Brown *et al*. 2003) and arsenical resistance operon repressor (ArsR) family transcriptional regulators (Wu and Rosen 1991) are related to heavy metal resistance. L-fucose isomerase is involved in the carbohydrate metabolism of bacteria (Seemann and Schulz 1997).

In the phylogenetic trees, some of these genes conflicted with their original lineages that were found in the generalist group orthologs. Conflicting trees suggests HGT events. For instance, there were conflicts for the XRE family transcriptional regulator, integral membrane protein PlnU, MerR family transcriptional regulator, L-fucose isomerase and the MarR family transcriptional regulator (Figure S1, Supporting Information).

### The network of orthologs shared by strains with high sugar utilization

**Figure 5.**
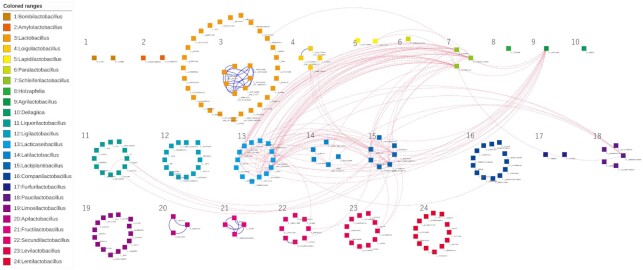
The networks for the generalist and specialist group orthologs. Each of the 178 nodes represents an LAB genome, which are colored and numbered by genus. Edges of dotted-red/solid-blue were created between two genomes when the number of sharing generalist/specialist group orthologs was more than five.

We constructed networks for the shared orthologs among the 178 strains in the 24 genera to identify the influence of sugar utilization on the GECs for different ecological niches (Fig. 5). There were 178 nodes to represent each genome, which were color-coded according to the 24 genera. An edge was generated between two genomes when they shared more than five orthologs of the generalist group or specialist group for sugar utilization. A dense network indicates that the community forms a GEC or has conserved genes inherited from their ancestors. There were no edges identified in this investigation among the following genera *Bombilactobacillus*, *Amylolactobacillus*, *Paralactobacillus*, *Holzapfelia*, *Dellaglioa*, *Furfurilactobacillus* and *Lentilactobacillus*.

While the networks of orthologs predominantly shared by the specialist groups for sugar utilization were connected only between the same genera, the networks of the generalist groups were connected across genera. The networks of specialists were made by strains from *Lactobacillus*, *Loigolactobacillus*, *Apilactobacillus*, *Fructilactobacillus* and *Secundilactobacillus* independently. The generalist networks connected *Lactobacillus*, *Loigolactobacillus*, *Lapidilactobacillus*, *Schleiferilactobacillus*, *Agrilactobacillus*, *Liquorilactobacillus*, *Lacticaseibacillus*, *Lactilactobacillus*, *Lactiplantibacillus*, *Companilactobacillus*, *Paucilactobacillus*, *Secundilactobacillus* and *Levilactobacillus*.

In the generalist networks, the edges were connected between distant strains isolated from similar environments. As a result of community extraction, the number of communities was 51, the maximum number of strains in the community was nine and the minimum value was two (Table S2, Supporting Information). Communities were often formed from groups of the following three genera, *Schleiferilactobacillus*, *Lacticaseibacillus* and *Lactiplantibacillus*, or four when *Agrilactobacillus* was added. For example, a community was formed by *Schleiferilactobacillus harbinensis*, *Schleiferilactobacillus perolens*, *Lactiplantibacillus paraplantarum*, *Lacticaseibacillus rhamnosus*, *L. casei* and *Agrilactobacillus composti* that were isolated from vegetables and brewing-related environments (Table S1, Supporting Information; Zheng *et al*. 2020). In addition, some communities amongst the genus *Lactiplantibacillus* and *Liquorilactobacillus* were identified. All members of a community between *Liquorilactobacillus nagelii*, *Lactiplantibacillus paraplantarum* and *Lactiplantibacillus plantarum* ssp. *plantarum*, were isolated from dairy products (Table S1, Supporting Information; Zheng *et al*. 2020).

The analysis method aimed to select high ASU value orthologs, and as a result, strains with low sugar utilization values tended not to be included in the generalist networks. For example, the genus *Lacticaseibacillus*, *L. nasuensis*, *L. thailandensis* and *L. pantheris* were not included in the generalist network, and neither were *L. nasuensis* and *L. thailandensis* which have small sugar utilization values. Moreover, for the genus *Latilactobacillus*, all strains except for *L. skei* ssp *carnosus* and *L. fuchuensis* had relatively low sugar utilization values and were not included in the network.

Despite this, the generalist network includes strains with low sugar utilization values. In these cases, the strains were connected to closely related strains with high values. For example, while *L. brantae* had a low sugar utilization value, it shared generalist group orthologs with *Schleiferilactobacillus harbinensis*, *Schleiferilactobacillus shenzhenensis* and *L. saniviri*. *L. brantae* is closely related to *L. saniviri* which had a high sugar utilization value. In addition, *Lactobacillus paracasei* and *L. paracasei* ssp. *Tolerans* were also included in the generalist network, although they had low sugar utilization values, and were closely related to *L. paracasei* ssp. *paracasei* which had a high sugar utilization value.

## DISCUSSION

In this study, we investigated the influence of genomic and phenotypic features on the construction of ecological GECs for *Lactobacillaceae* family. A total of six phenotypes and seven genomic features were investigated to identify which factors influenced HGT bias. Mapping the obtained data to a phylogenetic tree suggested that there were relationships between the phenotypes and genomic features (Fig. 1). Multiple regression analyses were performed to identify which genomic and phenotypic factors had the most significant effects on HGT (Fig. 2). The networks of orthologs were analyzed to identify how the phenotypes contributed to the formation of GECs (Fig. 5). These results suggested that the ability to utilize a variety of sugars contributed to increased HGT and the formation of GECs in the ecological niches among the genera. These results will help to improve our understanding of the evolution of related bacteria in ecological niches.

HGT tends to occur among prokaryotes that share similar phenotypes, as they live in the same environment (Jain *et al*. 2003). For example, many bacteria in the order Thermotogales of the *Thermotogae* which is composed mostly of thermophilic bacteria and in the class Clostridia which is included in the phylum Firmicutes, share ecological niches and genes, probably because they share thermophilic features (Andam and Gogarten 2011). These reports suggest that some phenotypes contribute to the sharing of ecological niches and the formation of GECs. Our study showed that this tendency can apply to bacterial groups within the *Lactobacillaceae* family and revealed that the utilization of a variety of sugars highly influenced the construction of GECs across genera to share niches such as vegetables, dairy and brewing-related environments (Fig. 5; Tables S1 and S2, Supporting Information).

The phenotypes for carbon utilization and oxygen tolerance were previously shown to influence HGT (Jain *et al*. 2003). The results of this investigation did not support this, however. Rather, sugar utilization value which means the number of sugar types that can be utilized was found to contribute to the formation of GECs. The sugar utilization values in this study differed from the carbon utilization feature that was defined heterotroph or autotroph in their previous study. The gaps of optimum conditions for growth in the laboratory and environment may hide possible effects on HGT (Jain *et al*. 2003). Moreover, as all lactic acid bacteria are heterotrophic organisms, we did not analyze this factor. In addition, there was no HGT that was related to oxygen tolerance, but there was a bias as approximately 80% of the strains in this study were facultatively anaerobic. That may have prevented the detection of a correlation between oxygen tolerance and HGT. The results of Jain *et al*. may thus be different because they investigated HGT across domains (empires), while we investigated HGT in the same family.

GECs among the strains of *Lactobacillaceae* family with high sugar utilization values could help to expand their habitats and promote the exchange of genetic material with various functions. According to our results for the functional classification by COG, there were a variety of gene functions in the generalist group orthologs for sugar utilization but the function proportions were not significantly different from those of the of the specialist group orthologs (Fig. 4). In the generalist group orthologs, there were not only genes related to sugar metabolism, but also genes to enable the habitation of various niches that were related to stress responses, bacteriocin production, antibiotic resistance, survival in the intestinal environment and heavy metal resistance. These results are consistent with the idea that most HGT genes are acquired with neutral or nearly neutral effects (Soucy *et al*. 2015). Some HGT genes in the GECs of different ecological niches may thus help recipients to adapt to new habitats, and affects population diversification (Baquero *et al*. 2021). These results allow us to speculate that the GECs composed of strains in *Lactobacillaceae* family with high sugar utilization accelerated their adaptations to new niches.

Overall, our results indicate that the phenotype to utilize a variety of sugars was the key factor for the construction of GECs in the family *Lactobacillaceae*. This feature is consistent with the fact that the *Lactobacillaceae* family contributes to the production of a wide variety of fermented foods by sharing niches such as vegetables, dairy products and brewing-related environments. The results of this study will help to improve our understanding of these ecologies.

## Supplementary Material

fnab117_Supplemental_Files
