# Supplementary material for: A sugar utilization phenotype contributes to the formation of genetic exchange communities in lactic acid bacteria
Source: FEMS Microbiol Lett. 2021 Sep 1;368(17):fnab117. doi: 10.1093/femsle/fnab117 (PMC8440127; doi:10.1093/femsle/fnab117)
Supplement: fnab117_Supplemental_Files [file fnab117_supplemental_files.zip › Supplementary_data_Table_S1.docx]

| accession number (id) | original name | strain | new name | type status | genome size (total sequence length) | number of CDS | G/C content | number of rRNA | number of tRNA | number of CRISPRS | number of CDS judged HGT | sugar utilization value (number of sugar types to be able to utilize) | growth at 15 | growth at 45 | micro aerophilic | facultatively anaerobic | anaerobic | isolation source |
| --- | --- | --- | --- | --- | --- | --- | --- | --- | --- | --- | --- | --- | --- | --- | --- | --- | --- | --- |
| SRR1151200 | Lactobacillus composti | DSM 18527 | Agrilactobacillus composti | type strain | 3463695 | 3306 | 44 | 2 | 51 | 3 | 80 | 11 | 1 | 0 | 0 | 1 | 0 | compost from shochu mash solids / pulque, a Mexican alcoholic beverage |
| SRR1151258 | Lactobacillus amylophilus | DSM 20533 | Amylolactobacillus amylophilus | type strain | 1546306 | 1550 | 43.7 | 0 | 52 | 1 | 126 | 3 | 1 | 0 | 0 | 1 | 0 | swine waste-corn fermentation / corn-starch processing industrial wastes / kocho (Ensete ventricosum) bread |
| ERR387486 | Lactobacillus amylotrophicus | DSM 20534 | Amylolactobacillus amylotrophicus | type strain | 1600645 | 1602 | 42.6 | 2 | 51 | 0 | 181 | 5 | 1 | 0 | 0 | 1 | 0 | corn silage |
| SRR1752129 | Lactobacillus apinorum | Fhon13 | Apilactobacillus apinorum | type strain | 1428890 | 1317 | 34.6 | 1 | 59 | 2 | 43 |  |  |  |  |  |  | honey stomach of the honeybee |
| GCA_001281265.1 | Lactobacillus kunkeei | YH-15 | Apilactobacillus kunkeei | type strain | 1515712 | 1353 | 36.4 | 3 | 62 | 0 | 54 | 2 | 1 | 0 | 0 | 1 | 0 | a sluggish grape wine fermentation / honey bees and flowers |
| ERR433479 | Lactobacillus ozensis | DSM 23829 | Apilactobacillus ozensis | type strain | 1476372 | 1439 | 31.9 | 3 | 56 | 2 | 42 | 0 | 1 | 0 | 0 | 0 | 1 | chrysanthemum flower |
| GCA_000970795.1 | Lactobacillus mellifer | Bin4 | Bombilactobacillus mellifer | type strain | 1815047 | 1661 | 39.3 | 3 | 50 | 0 | 139 |  |  |  |  |  |  | the honey stomach of the honeybee Apis mellifera |
| GCA_000967245.1 | Lactobacillus mellis | Hon2 | Bombilactobacillus mellis | type strain | 1810599 | 1650 | 36.2 | 3 | 53 | 0 | 55 |  |  |  |  |  |  | the honey stomach of the honeybee Apis mellifera |
| ERR387471 | Lactobacillus alimentarius | DSM 20249 | Companilactobacillus alimentarius | type strain | 2331920 | 2232 | 35.4 | 3 | 51 | 0 | 70 | 10 | 1 | 0 | 1 | 0 | 0 | marinated fish products / fermented sausages / ready-to-eat meats / type I sourdough / other plant fermentations |
| GCA_001438825.1 | Lactobacillus crustorum | LMG 23699 | Companilactobacillus crustorum | type strain | 2235695 | 2165 | 35 | 5 | 48 | 1 | 74 | 4 | 1 | 0 | 0 | 1 | 0 | sourdough / dairy products / forages |
| GCA_001434775.1 | Lactobacillus farciminis | DSM 20184 | Companilactobacillus farciminis | type strain | 2480845 | 2417 | 36.4 | 3 | 40 | 2 | 70 | 10 | 1 | 0 | 1 | 0 | 0 | meat products / sourdough / fermentend fish / cold-smoked salmon / soy sauce mash / dairy products / table olives / fermented vegetables / corn silage |
| ERR387495 | Lactobacillus futsaii | JCM 17355 | Companilactobacillus futsaii | type strain | 2490561 | 2449 | 35.6 | 2 | 51 | 1 | 88 | 9 | 1 | 0 | 0 | 1 | 0 | traditional fermented mustard products / fu-tsai and suan-tsai / it has been used experimentally for fermentation of shrimp waste |
| GCA_001050475.1 | Lactobacillus ginsenosidimutans | EMML 3141 | Companilactobacillus ginsenosidimutans | type strain | 2590556 | 2558 | 36.7 | 9 | 55 | 0 | 101 | 8 | 1 | 0 | 0 | 1 | 0 | kimchi |
| GCA_000831645.3 | Lactobacillus heilongjiangensis | DSM 28069 | Companilactobacillus heilongjiangensis | type strain | 2790548 | 2485 | 37.5 | 12 | 55 | 1 | 98 |  |  |  |  |  |  | fermented vegetables / type I sourdough |
| GCA_001438805.1 | Lactobacillus kimchiensis | DSM 24716 | Companilactobacillus kimchiensis | type strain | 2698724 | 2579 | 35.5 | 2 | 38 | 2 | 76 | 10 | 1 | 0 |  |  |  | kimchi |
| ERR387524 | Lactobacillus mindensis | DSM 14500 | Companilactobacillus mindensis | type strain | 2326589 | 2205 | 38.2 | 2 | 53 | 2 | 81 | 5 | 1 | 0 | 1 | 0 | 0 | type I sourdough |
| ERR433477 | Lactobacillus nantensis | DSM 16982 | Companilactobacillus nantensis | type strain | 2923132 | 2774 | 36.2 | 2 | 55 | 1 | 83 | 14 | 0 | 0 | 0 | 1 | 0 | type I sourdough |
| GCA_001435555.1 | Lactobacillus nodensis | DSM 19682 | Companilactobacillus nodensis | type strain | 2683197 | 2654 | 37.6 | 3 | 55 | 2 | 108 | 4 | 1 | 0 | 0 | 0 | 1 | fermented rice bran paste / it has been used experimentally as adjunct culture in cheese |
| SRR1151251 | Lactobacillus paralimentarius | DSM 13238 | Companilactobacillus paralimentarius | type strain | 2533817 | 2454 | 35.1 | 2 | 53 | 3 | 70 | 8 | 1 | 0 | 0 | 1 | 0 | sourdough / other cereal fermentations / poultry meat |
| ERR387549 | Lactobacillus tucceti | DSM 20183 | Companilactobacillus tucceti | type strain | 2170671 | 2102 | 34.1 | 3 | 56 | 1 | 56 | 5 | 1 | 0 | 1 | 0 | 0 | sausage |
| SRR1151144 | Lactobacillus versmoldensis | DSM 14857 | Companilactobacillus versmoldensis | type strain | 2386851 | 2344 | 38.3 | 3 | 55 | 1 | 104 | 5 | 1 | 0 |  |  |  | poultry salami |
| GCA_001434695.1 | Lactobacillus algidus | DSM 15638 | Dellaglioa algida | type strain | 1590323 | 1531 | 36 | 3 | 33 | 0 | 35 | 7 | 1 | 0 | 0 | 1 | 0 | refrigerated beef and pork meat |
| SRR1151205 | Lactobacillus florum | DSM 22689 | Fructilactobacillus florum | type strain | 1354760 | 1313 | 41.1 | 3 | 47 | 1 | 153 | 0 | 1 | 0 | 0 | 1 | 0 | peony / bietou flowers / grapes / wine |
| SRR1151190 | Lactobacillus fructivorans | DSM 20203 | Fructilactobacillus fructivorans | type strain | 1372674 | 1336 | 38.9 | 1 | 58 | 0 | 218 | 0 | 1 | 0 | 1 | 0 | 0 | the intestinal microbiota of fruit flies / spoiled sake mashes / spoiled mayonnaise / salad dressings / sour dough / dessert wines / aperitifs |
| GCA_001311115.1 | Lactobacillus lindneri | JCM 11027 | Fructilactobacillus lindneri | type strain | 1436854 | 1632 | 34.1 | 3 | 55 | 2 | 164 | 1 | 1 | 0 | 1 | 0 | 0 | spoiled beer / wine |
| SRR1151254 | Lactobacillus sanfranciscensis | DSM 20451 | Fructilactobacillus sanfranciscensis | type strain | 1253219 | 1278 | 34.7 | 4 | 57 | 1 | 103 | 1 | 1 | 0 | 0 | 1 | 0 | traditional sourdoughs / agave mash |
| GCA_000428925.1 | Lactobacillus rossiae | DSM 15814 | Furfurilactobacillus rossiae | type strain | 2862294 | 2700 | 43.3 | 5 | 58 | 1 | 89 | 4 | 1 | 0 |  |  |  | wheat sourdough / related cereal fermentations / beer / fruit / fecal samples of children and swine / it was used experimentally as starter culture for cactus pear fermentation [243] |
| ERR387541 | Lactobacillus siliginis | DSM 22696 | Furfurilactobacillus siliginis | type strain | 2041760 | 1980 | 44.1 | 1 | 53 | 0 | 118 | 4 | 0 | 0 | 0 | 1 | 0 | wheat sourdough |
| ERR387489 | Lactobacillus floricola | DSM 23037 | Holzapfelia floricola | type strain | 1287117 | 1247 | 34.5 | 3 | 44 | 3 | 33 | 0 | 1 | 0 | 0 | 1 | 0 | flowers |
| GCA_001436115.1 | Lactobacillus brantae | DSM 23927 | Lacticaseibacillus brantae | type strain | 1929842 | 1900 | 47.5 | 3 | 27 | 2 | 46 | 4 | 0 | 0 |  |  |  | the faeces of wild Canada goose (Branta canadensis) / experimental sourdoughs |
| ERR387469 | Lactobacillus camelliae | DSM 22697 | Lacticaseibacillus camelliae | type strain | 2553708 | 2403 | 55.4 | 0 | 51 | 0 | 259 | 9 | 0 | 0 |  |  |  | fermented tea (Camellia sinensis) leaves / fermented tomato pomace |
| GCA_000829055.1 | Lactobacillus casei | ATCC 393 | Lacticaseibacillus casei | type strain | 2952961 | 2890 | 47.9 | 15 | 59 | 0 | 269 | 14 | 1 | 0 |  |  |  | chinese traditional pickle / infant faeces / corn liquor / oat silage / commercial dietary supplements / sputum / nasopharynx |
| GCA_001433745.1 | Lactobacillus zeae | DSM 20178 | Lacticaseibacillus casei | type strain | 3121340 | 2961 | 47.7 | 5 | 53 | 3 | 54 | 14 | 1 | 0 |  |  |  | chinese traditional pickle / infant faeces / corn liquor / oat silage / commercial dietary supplements / sputum / nasopharynx |
| ERR387460 | Lactobacillus manihotivorans | DSM 13343 | Lacticaseibacillus manihotivorans | type strain | 3081436 | 3012 | 47.7 | 2 | 50 | 0 | 272 | 12 | 1 | 1 | 0 | 1 | 0 | sour cassava starch fermentation / tomato pomace silage |
| SRR1151220 | Lactobacillus nasuensis | JCM 17158 | Lacticaseibacillus nasuensis | type strain | 2278732 | 2137 | 57 | 6 | 64 | 0 | 160 | 2 | 0 | 0 | 0 | 1 | 0 | Sudan grass [Sorghum sudanense (Piper) Stapf] silage |
| ERR387506 | Lactobacillus pantheris | DSM 15945 | Lacticaseibacillus pantheris | type strain | 2531803 | 2293 | 52.9 | 2 | 52 | 0 | 228 | 8 | 1 | 0 | 0 | 1 | 0 | the faeces of a jaguar in Beijing Zoo / fermented vegetables |
| GCA_000014525.1 | Lactobacillus paracasei | ATCC 334 | Lacticaseibacillus paracasei |  | 2924325 | 2835 | 46.6 | 15 | 60 | 1 | 180 | 4 | 1 | 0 |  |  |  | a variety of courses including the human oral cavity / fermented cereals / vegetables / meats / dairy products / invertebrate hosts |
| GCA_000829035.1 | Lactobacillus paracasei subsp. paracasei | JCM 8130 | Lacticaseibacillus paracasei ssp. paracasei | type strain | 3017804 | 2945 | 46.6 | 15 | 62 | 0 | 226 | 13 | 1 | 0 |  |  |  | dairy products / sewage / silage / humans and clinical sources |
| SRR1151129 | Lactobacillus paracasei subsp. tolerans | DSM 20258 | Lacticaseibacillus paracasei ssp. tolerans | type strain | 2413718 | 2419 | 46.4 | 1 | 46 | 0 | 185 | 3 | 1 | 0 |  |  |  | dairy products / tomato pomace silage |
| SRR1151270 | Lactobacillus rhamnosus | DSM 20021 | Lacticaseibacillus rhamnosus | type strain | 2945929 | 2738 | 46.7 | 3 | 56 | 0 | 138 | 15 | 1 | 1 |  |  |  | a broad range of habitats including dairy products / fermented meat / fish / vegetables and cereals / sewage / humans (oral, vaginal and intestinal) / invertebrate hosts and clinical sources |
| ERR433494 | Lactobacillus saniviri | DSM 24301 | Lacticaseibacillus saniviri | type strain | 2429351 | 2409 | 47.7 | 1 | 56 | 1 | 184 | 14 | 1 | 0 | 0 | 1 | 0 | the faeces of a healthy man / fermented rice / fermented fish |
| ERR387540 | Lactobacillus sharpeae | DSM 20505 | Lacticaseibacillus sharpeae | type strain | 2438466 | 2344 | 53.4 | 3 | 50 | 0 | 342 | 7 | 1 | 0 |  |  |  | municipal sewage / spoiled meat |
| SRR1151237 | Lactobacillus thailandensis | DSM 22698 | Lacticaseibacillus thailandensis | type strain | 2064913 | 1893 | 53.5 | 1 | 52 | 1 | 154 | 4 | 0 | 0 |  |  |  | fermented fish (pla-ra) in Thailand |
| ERR387476 | Lactobacillus fabifermentans | DSM 21115 | Lactiplantibacillus fabifermentans | type strain | 3271316 | 3111 | 45 | 2 | 60 | 0 | 156 | 5 | 1 | 0 | 0 | 1 | 0 | cocoa bean heap fermentation / fermented grapes / fermented cereals |
| GCA_001039045.1 | Lactobacillus herbarum | TCF032-E4 | Lactiplantibacillus herbarum | type strain | 2899876 | 2805 | 43.5 | 4 | 36 | 0 | 184 |  |  |  |  |  |  | fermented radish |
| GCA_001435655.1 | Lactobacillus paraplantarum | DSM 10667 | Lactiplantibacillus paraplantarum | type strain | 3395753 | 3192 | 43.7 | 0 | 26 | 0 | 231 | 15 | 1 | 0 | 0 | 1 | 0 | beer / human faeces / grape marmalade / dairy products / jangajji, Korean fermented food / fermented vegetables / fermented fruits / fermented dates / rice bran pickles / silage / cocoa beans / fermented sourdough / fermented slurry / faecal microbita of healthy dogs / traditional fura processing / wine / sow milk |
| SRR1151242 | Lactobacillus pentosus | DSM 20314 | Lactiplantibacillus pentosus | type strain | 3642579 | 3285 | 46.3 | 4 | 68 | 5 | 188 | 17 | 1 | 0 | 0 | 1 | 0 | diverse sources including corn silage / fermenting olives / sewage / fermented mulberry leaf powders / fermented teas / glutinous rice dough / corn noodles / chili sauce / mustard pickles / stinky tofu / dairy products / mustard pickle / fermented idli batter / tempoyak / human vagina / human stools / sourdoughs |
| ERR387522 | Lactobacillus plantarum subsp. argentoratensis | DSM 16365 | Lactiplantibacillus plantarum ssp. argentoratensis | type strain | 3172036 | 2939 | 45 | 0 | 46 | 3 | 181 | 17 | 1 | 0 | 0 | 1 | 0 | starchy food / fermenting food of plant origin / timothy / orchardgrass and elephant grass silage / fermented Uttapam batter / fermented idli batter |
| SRR1151193 | Lactobacillus plantarum subsp. plantarum | CGMCC 1.2437 | Lactiplantibacillus plantarum ssp. plantarum | type strain | 3220167 | 3019 | 44.5 | 4 | 63 | 0 | 208 | 17 | 1 | 0 | 0 | 1 | 0 | dairy products and dairy environments / silage / sauerkraut / pickled vegetables / sourdough / cow dung / the human mouth / intestinal tract and stools / sewage |
| GCA_001438845.1 | Lactobacillus xiangfangensis | LMG 26013 | Lactiplantibacillus xiangfangensis | type strain | 2989578 | 2757 | 45.1 | 4 | 50 | 0 | 162 | 12 | 0 | 0 | 0 | 1 | 0 | pickle / sourdough |
| ERR387527 | Lactobacillus acetotolerans | DSM 20749 | Lactobacillus acetotolerans | type strain | 1571585 | 1518 | 36.2 | 3 | 55 | 3 | 65 | 3 | 0 | 0 | 0 | 1 | 0 | mash fermenta tions for production of grain liquor and vinegar in China and Japan / plant fermentations / silage / intestine of swine / ducks / cattle |
| GCA_000786395.1 | Lactobacillus acidophilus | ATCC 4356 | Lactobacillus acidophilus | type strain | 1956698 | 1884 | 34.6 | 4 | 55 | 1 | 61 | 9 | 0 | 1 | 1 | 0 | 0 | intestinal tract of humans and animals / human mouth / human vagina / sourdough / wine |
| SRR1151257 | Lactobacillus amylolyticus | DSM 11664 | Lactobacillus amylolyticus | type strain | 1539298 | 1574 | 38.3 | 3 | 55 | 0 | 84 | 4 | 0 | 1 | 1 | 0 | 0 | malt / mash and unhopped wort in breweries / sourdough / tofu whey |
| GCA_001433985.1 | Lactobacillus amylovorus | DSM 20531 | Lactobacillus amylovorus | type strain | 2017377 | 2045 | 37.8 | 4 | 36 | 0 | 235 | 9 | 0 | 1 | 0 | 1 | 0 | swine intestinal / sourdough / cattle waste-corn fermentation |
| GCA_000970735.1 | Lactobacillus apis | Hma11 | Lactobacillus apis |  | 1717379 | 1564 | 36.6 | 3 | 50 | 1 | 19 |  |  |  |  |  |  | stomach contents of honeybees |
| GCA_001434005.1 | Lactobacillus crispatus | DSM 20584 | Lactobacillus crispatus | type strain | 2057071 | 2017 | 36.6 | 3 | 43 | 1 | 79 | 9 | 1 | 1 | 0 | 1 | 0 | human faeces / vagina and buccal cavities / crops and caeca of chicken / patients with purulent pleurisy / leucorrhea and urinary tract infections / type II sourdoughs |
| GCA_000056065.1 | Lactobacillus delbrueckii subsp. bulgaricus | ATCC 11842 | Lactobacillus delbrueckii ssp. bulgaricus | type strain | 1864998 | 1900 | 49.7 | 27 | 95 | 1 | 122 | 1 | 0 | 1 |  |  |  | yoghurt / cheese / intestinal microbiota of suckling piglets |
| GCA_001263315.1 | Lactobacillus delbrueckii subsp. delbrueckii | KACC 13439 | Lactobacillus delbrueckii ssp. delbrueckii | type strain | 1766190 | 1769 | 50 | 1 | 50 | 0 | 48 | 2 | 0 | 1 |  |  |  | vegetable source / sour grain mash / fermented grains |
| GCA_001189855.1 | Lactobacillus delbrueckii subsp. indicus | JCM 15610 | Lactobacillus delbrueckii ssp. indicus | type strain | 1877412 | 1832 | 49.5 | 7 | 64 | 1 | 158 | 2 | 0 | 1 |  |  |  | a fermented dairy product dahi from India |
| GCA_000387565.1 | Lactobacillus delbrueckii subsp. jakobsenii | ZN7a-9 | Lactobacillus delbrueckii ssp. jakobsenii | type strain | 1730812 | 1677 | 50.2 | 3 | 45 | 2 | 100 | 2 | 0 | 1 |  |  |  | dolo wort used in the production of the fermented African beverge dolo in Burkina Faso |
| GCA_000192165.1 | Lactobacillus delbrueckii subsp. lactis | DSM 20072 | Lactobacillus delbrueckii ssp. lactis | type strain | 2071079 | 1864 | 49.8 | 3 | 72 | 1 | 180 | 0 | 0 | 1 | 0 | 1 | 0 | milk / cheese / compressed yeasts / grain mash |
| GCA_001190005.1 | Lactobacillus delbrueckii subsp. sunkii | JCM 17838 | Lactobacillus delbrueckii ssp. sunkii | type strain | 1945263 | 1823 | 50.1 | 9 | 74 | 2 | 128 | 11 | 0 | 0 | 0 | 1 | 0 | a traditionally fermented Japanese red turnip |
| GCA_001434815.1 | Lactobacillus equicursoris | DSM 19284 | Lactobacillus equicursoris | type strain | 2052598 | 1873 | 47.7 | 3 | 28 | 2 | 143 | 6 | 0 | 1 | 0 | 0 | 1 | a thoroughbred racehorse |
| ERR387508 | Lactobacillus gallinarum | DSM 10532 | Lactobacillus gallinarum | type strain | 1925768 | 1912 | 36.5 | 3 | 58 | 0 | 94 | 10 | 1 | 1 | 0 | 1 | 0 | chicken intestine |
| GCA_000014425.1 | Lactobacillus gasseri | ATCC 33323 | Lactobacillus gasseri | type strain | 1894360 | 1808 | 35.3 | 18 | 78 | 0 | 55 | 7 | 0 | 1 | 0 | 0 | 1 | human female lower genital tract / the human mouth / intes tinal tract / the intestine of animals / wounds, urine, blood, carious dentine and pus of patients suffering from septic infections |
| SRR1151155 | Lactobacillus gigeriorum | DSM 23908 | Lactobacillus gigeriorum | type strain | 1906781 | 1870 | 37 | 2 | 56 | 0 | 63 | 8 | 0 | 1 | 0 | 1 | 0 | a crop of a chicken |
| ERR387507 | Lactobacillus hamsteri | DSM 5661 | Lactobacillus hamsteri | type strain | 1790730 | 1712 | 35.1 | 3 | 58 | 2 | 66 | 14 | 0 | 0 | 0 | 0 | 1 | the intestine of a hamster |
| GCA_000970855.1 | Lactobacillus helsingborgensis | Bma5 | Lactobacillus helsingborgensis | type strain | 2020254 | 1823 | 36.3 | 3 | 51 | 2 | 101 |  |  |  |  |  |  | the honey stomach of the honeybee A. mellifera mellifera / alfalfa silage |
| GCA_000160855.1 | Lactobacillus helveticus | DSM 20075 | Lactobacillus helveticus | type strain | 2020582 | 1944 | 36.8 | 3 | 37 | 1 | 177 | 2 | 0 | 1 | 0 | 1 | 0 | chicken / sour milk / cheese starter cultures and cheese / particularly Emmental and Gruye?re cheeses / tomato pomace / silage |
| SRR1151158 | Lactobacillus hominis | DSM 23910 | Lactobacillus hominis | type strain | 1930068 | 1882 | 35.2 | 2 | 56 | 1 | 66 | 10 | 0 | 1 | 0 | 1 | 0 | the human intestine |
| GCA_000160875.1 | Lactobacillus iners | DSM 13335 | Lactobacillus iners | type strain | 1277649 | 1191 | 32.5 | 3 | 45 | 0 | 72 | 0 | 0 | 0 | 0 | 1 | 0 | the human female lower genital tract / human skin |
| ERR387510 | Lactobacillus intestinalis | DSM 6629 | Lactobacillus intestinalis | type strain | 1993045 | 1838 | 35.3 | 3 | 52 | 5 | 53 | 5 | 0 | 1 | 0 | 1 | 0 | the intestines of rats, mice and pigs |
| SRR1151162 | Lactobacillus jensenii | DSM 20557 | Lactobacillus jensenii | type strain | 1615929 | 1478 | 34.3 | 3 | 39 | 1 | 47 | 10 | 0 | 1 | 0 | 1 | 0 | the human female lower genital tract. |
| SRR1151139 | Lactobacillus johnsonii | ATCC 33200 | Lactobacillus johnsonii | type strain | 1770443 | 1767 | 34.4 | 3 | 52 | 0 | 41 | 8 | 1 | 1 | 0 | 0 | 1 | humans (gut, vagina) / the faeces of birds / rodents / calves and pigs / type II sourdoughs |
| SRR1151163 | Lactobacillus kalixensis | DSM 16043 | Lactobacillus kalixensis | type strain | 2073352 | 1935 | 36.1 | 3 | 62 | 1 | 81 | 12 | 0 | 1 |  |  |  | a biopsy of the healthy human gastric mucosa |
| SRR1151211 | Lactobacillus kefiranofaciens subsp. kefiranofaciens | DSM 5016 | Lactobacillus kefiranofaciens ssp. kefiranofaciens | type strain | 2258515 | 2316 | 37.3 | 3 | 58 | 2 | 150 | 7 | 0 | 0 | 0 | 1 | 0 | kefir grains / fermented dairy products |
| SRR1151212 | Lactobacillus kefiranofaciens subsp. kefirgranum | DSM 10550 | Lactobacillus kefiranofaciens ssp. kefirgranum | type strain | 2084861 | 2099 | 37.5 | 2 | 58 | 4 | 136 | 5 | 0 | 0 | 0 | 1 | 0 | kefir grains |
| GCA_000970755.1 | Lactobacillus kimbladii | Hma2 | Lactobacillus kimbladii | type strain | 2186983 | 1972 | 35.8 | 3 | 50 | 2 | 100 |  |  |  |  |  |  | the honey stomach of the honeybee A. mellifera |
| ERR387512 | Lactobacillus kitasatonis | DSM 16761 | Lactobacillus kitasatonis | type strain | 1906076 | 1917 | 37.5 | 2 | 59 | 0 | 153 | 5 | 0 | 1 | 0 | 1 | 0 | the intestine of animals including chicken / swine |
| SRR1745849 | Lactobacillus kullabergensis | Biut2 | Lactobacillus kullabergensis | type strain | 2080753 | 1939 | 35.5 | 3 | 53 | 1 | 92 |  |  |  |  |  |  | the honey stomach of the honeybee A. mellifera mellifera |
| GCA_000970775.1 | Lactobacillus melliventris | Hma8 | Lactobacillus melliventris | type strain | 2116151 | 1994 | 35.8 | 3 | 51 | 1 | 106 |  |  |  |  |  |  | the homey stomach of honeybees |
| SRR1151169 | Lactobacillus pasteurii | DSM 23907 | Lactobacillus pasteurii | type strain | 1753652 | 1684 | 38.5 | 1 | 54 | 1 | 66 | 10 | 0 | 1 | 0 | 1 | 0 | the human intestine |
| ERR387520 | Lactobacillus psittaci | DSM 15354 | Lactobacillus psittaci | type strain | 1542511 | 1344 | 35.7 | 3 | 52 | 1 | 29 | 2 | 1 | 1 | 0 | 1 | 0 | a hyacinth macaw |
| ERR387546 | Lactobacillus taiwanensis | DSM 21401 | Lactobacillus taiwanensis | type strain | 1865395 | 1816 | 33.9 | 2 | 52 | 0 | 38 | 7 | 0 | 1 | 0 | 1 | 0 | the mouse gastrointestinal tract / silage cattle feed |
| SRR1151174 | Lactobacillus ultunensis | DSM 16047 | Lactobacillus ultunensis | type strain | 2169096 | 2115 | 36 | 3 | 60 | 0 | 90 | 9 | 0 | 0 | 0 | 1 | 0 | a biopsy of a healthy human gastric mucosa |
| SRR1151132 | Lactobacillus concavus | DSM 17758 | Lapidilactobacillus concavus | type strain | 1903092 | 1765 | 43.3 | 1 | 49 | 1 | 77 | 5 | 0 | 0 | 0 | 1 | 0 | the walls of a distilled-spirit-fermenting cellar in China |
| SRR1151201 | Lactobacillus dextrinicus | DSM 20335 | Lapidilactobacillus dextrinicus | type strain | 1807580 | 1725 | 38 | 3 | 49 | 2 | 86 |  |  |  |  |  |  | silage / fermenting vegetables / beer / sliced vacuum-packed cooked sausage |
| SRR1151125 | Lactobacillus curvatus | DSM 20019 | Latilactobacillus curvatus | type strain | 1807340 | 1814 | 42 | 0 | 37 | 2 | 111 | 3 | 1 | 0 | 0 | 1 | 0 | cow dung / fermented and vacuum-packaged refrigerated meat / fermented and vacuum-packaged refrigerated fish / dairy products such as milk and cheese / fermented plant products like sauerkraut / sourdough (including prepacked finished dough and pressed yeast) / radish / pickles / kimchi / other plant derived materials like honey / the environmental fermentation process of corn or grass silage |
| GCA_000615805.1 | Lactobacillus fuchuensis | JCM 11249 | Latilactobacillus fuchuensis | type strain | 2107444 | 2205 | 41.8 | 3 | 34 | 1 | 78 | 11 | 1 | 0 |  |  |  | vacuum-packaged refrigerated beef / common carp intestine / other seafood products |
| ERR387528 | Lactobacillus graminis | DSM 20719 | Latilactobacillus graminis | type strain | 1829440 | 1739 | 40.3 | 2 | 51 | 1 | 95 | 5 | 1 | 0 |  |  |  | grass silage / meat products / sourdough / gut of snail Cornum aspersum / grapes |
| ERR433493 | Lactobacillus sakei subsp. carnosus | DSM 15831 | Latilactobacillus sakei ssp. carnosus | type strain | 1975630 | 1984 | 41 | 0 | 49 | 2 | 137 | 9 | 1 | 0 |  |  |  | fermented meat products / vacuum-packaged meat / sauerkraut / other fermented plant material |
| SRR1151267 | Lactobacillus sakei subsp. sakei | DSM 20017 | Latilactobacillus sakei ssp. sakei | type strain | 1907928 | 1891 | 41.1 | 0 | 51 | 0 | 87 | 7 | 1 | 0 |  |  |  | sake starter / fermented meat products / vacuum packaged meat / sauerkraut / other fermented plant material / human faeces |
| SRR1151196 | Lactobacillus buchneri | DSM 20057 | Lentilactobacillus buchneri | type strain | 2451635 | 2345 | 44.4 | 3 | 61 | 1 | 68 | 6 | 1 | 0 | 0 | 1 | 0 | pressed yeast / milk / cheese / fermenting plant material / the human mouth / used commercially as silage inoculant |
| GCA_000785105.1 | Lactobacillus curieae | CCTCC M 2011381 | Lentilactobacillus curieae | type strain | 2185962 | 2112 | 39.6 | 6 | 56 | 1 | 73 |  |  |  |  |  |  | stinky tofu brine / cocoa bean fermentations / cheese curd powder |
| ERR387480 | Lactobacillus diolivorans | DSM 14421 | Lentilactobacillus diolivorans | type strain | 3202031 | 2962 | 40 | 0 | 43 | 2 | 67 |  |  |  |  |  |  | maize silage / vegetable (cucumber) fermentations / fermented dairy products |
| GCA_001435875.1 | Lactobacillus farraginis | DSM 18382 | Lentilactobacillus farraginis | type strain | 2859511 | 2749 | 42 | 3 | 40 | 2 | 74 | 8 | 1 | 1 | 0 | 1 | 0 | a compost of distilled shochu residue |
| SRR1151260 | Lactobacillus hilgardii | DSM 20176 | Lentilactobacillus hilgardii | type strain | 2605214 | 2538 | 39.6 | 0 | 59 | 1 | 48 | 4 | 1 | 0 | 0 | 1 | 0 | spoiled wine / kefir grains / mezcal fermentations / silage |
| ERR387463 | Lactobacillus kefiri | DSM 20587 | Lentilactobacillus kefiri | type strain | 2322665 | 2208 | 41.7 | 1 | 57 | 3 | 61 | 4 | 1 | 0 |  |  |  | kefir as part of the core microbiota |
| SRR1151216 | Lactobacillus kisonensis | DSM 19906 | Lentilactobacillus kisonensis | type strain | 3017560 | 2765 | 41.8 | 0 | 58 | 4 | 55 | 6 | 1 | 0 | 0 | 1 | 0 | pickle brine |
| GCA_001434145.1 | Lactobacillus otakiensis | DSM 19908 | Lentilactobacillus otakiensis | type strain | 2346188 | 2255 | 42.4 | 3 | 28 | 2 | 59 | 7 | 1 | 0 | 0 | 1 | 0 | sunki, a fermented turnip product / kefir |
| SRR1151168 | Lactobacillus parabuchneri | DSM 5707 | Lentilactobacillus parabuchneri | type strain | 2568303 | 2377 | 43.4 | 1 | 58 | 4 | 81 | 10 | 1 | 0 | 0 | 1 | 0 | dairy products / saliva / silage / spoiled beer / some strains were shown to persist over month in whiskey mashes in Scottish distilleries |
| GCA_001435895.1 | Lactobacillus parafarraginis | DSM 18390 | Lentilactobacillus parafarraginis | type strain | 3081674 | 2921 | 45.2 | 3 | 52 | 5 | 78 | 9 | 0 | 0 | 0 | 1 | 0 | compost of distilled shochu residue / silage / fermented vegetables / kefir grains |
| SRR1151230 | Lactobacillus rapi | DSM 19907 | Lentilactobacillus rapi | type strain | 2848015 | 2645 | 42.9 | 0 | 57 | 3 | 40 | 10 | 1 | 0 | 0 | 1 | 0 | sunki / other vegetable fermentations |
| GCA_001436555.1 | Lactobacillus senioris | DSM 24302 | Lentilactobacillus senioris | type strain | 1566789 | 1568 | 39.1 | 3 | 44 | 0 | 46 | 4 | 1 | 0 | 0 | 1 | 0 | the faeces of a 100-year-old female |
| SRR1151235 | Lactobacillus sunkii | DSM 19904 | Lentilactobacillus sunkii | type strain | 2693190 | 2545 | 42.1 | 1 | 58 | 0 | 71 | 7 | 1 | 0 | 0 | 1 | 0 | sunki, a fermented turnip product / kefir |
| ERR387483 | Lactobacillus acidifarinae | DSM 19394 | Levilactobacillus acidifarinae | type strain | 2913834 | 2738 | 51.6 | 1 | 57 | 7 | 199 | 6 | 1 | 0 | 0 | 1 | 0 | type I wheat sourdough / fermented rice bran |
| GCA_001433855.1 | Lactobacillus brevis | DSM 20054 | Levilactobacillus brevis | type strain | 2474438 | 2423 | 46 | 4 | 42 | 0 | 84 | 6 | 1 | 0 | 0 | 1 | 0 | milk / cheese / sauerkraut and rrelated vegetable fermentations / sourdough / silage / cow manure / faeces / the mouth and intestinal tract of humans and rats |
| GCA_000807975.1 | Lactobacillus brevis | BSO 464 | Levilactobacillus brevis |  | 2723202 | 2700 | 45.4 | 18 | 48 | 1 | 149 | 6 | 1 | 0 | 0 | 1 | 0 | milk / cheese / sauerkraut and rrelated vegetable fermentations / sourdough / silage / cow manure / faeces / the mouth and intestinal tract of humans and rats |
| ERR387482 | Lactobacillus hammesii | DSM 16381 | Levilactobacillus hammesii | type strain | 2807716 | 2591 | 49.4 | 2 | 52 | 3 | 151 | 7 | 1 | 0 | 0 | 1 | 0 | wheat and rye sourdoughs / ryegrass silages / a municipal biogas plant |
| SRR1151217 | Lactobacillus koreensis | JCM 16448 | Levilactobacillus koreensis | type strain | 2940897 | 2666 | 49.2 | 1 | 55 | 2 | 171 | 5 | 1 | 0 | 0 | 1 | 0 | cabbage kimchi / sourdough |
| ERR433476 | Lactobacillus namurensis | DSM 19117 | Levilactobacillus namurensis | type strain | 2470988 | 2227 | 52 | 1 | 58 | 5 | 25 | 7 | 1 | 0 | 0 | 1 | 0 | wheat sourdough / vegetable fermentations |
| SRR1151225 | Lactobacillus parabrevis | ATCC 53295 | Levilactobacillus parabrevis | type strain | 2625389 | 2379 | 49 | 0 | 51 | 1 | 142 | 5 | 1 | 0 | 0 | 1 | 0 | farmhouse red Cheshire cheese / wheat sourdough / fermented vegetables / a municipal biogas plant |
| GCA_001437125.1 | Lactobacillus paucivorans | DSM 22467 | Levilactobacillus paucivorans | type strain | 2362603 | 2210 | 49.1 | 3 | 45 | 2 | 149 | 2 | 1 | 0 | 0 | 0 | 1 | storage tank of a brewery |
| GCA_001436675.1 | Lactobacillus senmaizukei | DSM 21775 | Levilactobacillus senmaizukei | type strain | 2222963 | 2122 | 48.6 | 2 | 61 | 1 | 107 | 5 | 1 | 0 | 0 | 1 | 0 | senmaizuke / a fermented turnip product |
| ERR387543 | Lactobacillus spicheri | DSM 15429 | Levilactobacillus spicheri | type strain | 2742678 | 2451 | 55.9 | 0 | 46 | 4 | 35 | 3 | 1 | 0 | 0 | 1 | 0 | wheat and rice sourdoughs / fermented vegetables / a municipal biogas plant |
| SRR1151256 | Lactobacillus zymae | DSM 19395 | Levilactobacillus zymae | type strain | 2700869 | 2444 | 53.6 | 3 | 63 | 5 | 38 | 4 | 1 | 0 | 0 | 1 | 0 | type I wheat sourdough / forages / fermented onions |
| GCA_001435755.1 | Lactobacillus acidipiscis | DSM 15836 | Ligilactobacillus acidipiscis | type strain | 2326083 | 2230 | 39.1 | 2 | 32 | 1 | 79 | 4 | 0 | 0 | 1 | 0 | 0 | fermented fish (pla-ra and pla-chom) in Thai land but also found in dairy products / soy sauce mash / table olives / sake starter / tropical grasses / forage crops / bee pollen / Chinese DaQu / a saccharification starter for production of vinegar / liquor from cereals |
| ERR387498 | Lactobacillus agilis | DSM 20509 | Ligilactobacillus agilis | type strain | 2047633 | 2015 | 41.7 | 0 | 63 | 3 | 176 | 15 | 0 | 1 | 0 | 1 | 0 | municipal sewage / the pigeon crops / the gut and cecum of birds / human gut and vagina / porcine intestinal mucin / Nigerian ogi / cheese / fermented food products such as masau fruits |
| ERR387553 | Lactobacillus animalis | DSM 20602 | Ligilactobacillus animalis | type strain | 1870553 | 1812 | 41.1 | 1 | 51 | 2 | 88 | 8 | 0 | 1 | 0 | 1 | 0 | dental plaques / intestines of animals |
| ERR433462 | Lactobacillus apodemi | DSM 16634 | Ligilactobacillus apodemi | type strain | 2082063 | 2019 | 38.6 | 3 | 52 | 3 | 163 | 9 | 0 | 1 | 0 | 1 | 0 | the faeces of a wild mouse |
| ERR438946 | Lactobacillus aviarius subsp. araffinosus | DSM 20653 | Ligilactobacillus araffinosus | type strain | 1470053 | 1410 | 38.1 | 2 | 46 | 0 | 66 | 4 | 0 | 0 | 0 | 1 | 0 | the intestine and faeces of birds |
| ERR387530 | Lactobacillus aviarius subsp. aviarius | DSM 20655 | Ligilactobacillus aviarius | type strain | 1674521 | 1585 | 40.1 | 0 | 43 | 0 | 109 | 7 | 0 | 0 | 0 | 1 | 0 | the intestine and faeces of birds |
| GCA_000423245.1 | Lactobacillus ceti | DSM 22408 | Ligilactobacillus ceti | type strain | 1385752 | 1269 | 33.7 | 5 | 37 | 0 | 118 | 1 | 1 | 0 | 0 | 1 | 0 | the lungs of a beaked whale |
| GCA_001435735.1 | Lactobacillus equi | DSM 15833 | Ligilactobacillus equi | type strain | 2284210 | 2188 | 39 | 2 | 50 | 4 | 211 | 6 | 0 | 1 | 0 | 1 | 0 | faeces of horses |
| ERR387461 | Lactobacillus hayakitensis | DSM 18933 | Ligilactobacillus hayakitensis | type strain | 1636658 | 1543 | 34 | 3 | 68 | 0 | 69 | 7 | 0 | 1 | 0 | 1 | 0 | the faeces of a thoroughbred as predominant species in the intestinal microbiota |
| ERR387504 | Lactobacillus murinus | DSM 20452 | Ligilactobacillus murinus | type strain | 2159096 | 2030 | 40 | 2 | 55 | 0 | 137 | 8 | 0 | 1 | 0 | 1 | 0 | the intestinal tract of mice and rats / sourdough |
| SRR1151229 | Lactobacillus pobuzihii | KCTC 13174 | Ligilactobacillus pobuzihii | type strain | 2332525 | 2124 | 37.7 | 0 | 59 | 0 | 48 | 6 | 0 | 0 | 0 | 1 | 0 | pobuzihi / fermented cummincordia / fermented fish / traditional vinegar |
| ERR433499 | Lactobacillus ruminis | ATCC 27780 | Ligilactobacillus ruminis | type strain | 2025861 | 1903 | 43.4 | 1 | 58 | 2 | 133 | 12 | 0 | 0 | 0 | 0 | 1 | rumen of cow and from sewage / horses and pigs and bovine uterus / the gut of humans |
| GCA_000423265.1 | Lactobacillus saerimneri | DSM 16049 | Ligilactobacillus saerimneri | type strain | 1720753 | 1726 | 42.5 | 4 | 35 | 0 | 132 | 3 | 0 | 1 | 0 | 1 | 0 | pig faeces / the intestines of pigs / the human gut and vagina / the cecum of chicken |
| GCA_000159395.1 | Lactobacillus salivarius | ATCC 11741 | Ligilactobacillus salivarius | type strain | 2017251 | 1929 | 32.6 | 3 | 37 | 1 | 89 | 9 | 0 | 1 | 0 | 1 | 0 | the mouth and intestinal tract of humans / cats / hamsters / chickens / dairy products / swine |
| SRR1151148 | Lactobacillus antri | DSM 16041 | Limosilactobacillus antri | type strain | 2249658 | 2113 | 51.1 | 2 | 61 | 2 | 239 | 7 | 0 | 1 | 0 | 0 | 1 | biopsy of a healthy human gastric mucosa / the intestine of other vertebrate animals |
| SRR1151152 | Lactobacillus coleohominis | DSM 14060 | Limosilactobacillus coleohominis | type strain | 1865893 | 1979 | 41.1 | 3 | 62 | 0 | 100 | 1 | 0 | 1 | 0 | 1 | 0 | the human vagina / in human intestinal microbiota / swine |
| SRR1151138 | Lactobacillus equigenerosi | DSM 18793 | Limosilactobacillus equigenerosi | type strain | 1599169 | 1545 | 42.7 | 1 | 60 | 0 | 82 | 4 | 0 | 1 | 0 | 1 | 0 | the intestinal tract of a thoroughbred horse |
| ERR203996 | Lactobacillus fermentum | ATCC 14931 | Limosilactobacillus fermentum | type strain | 1782450 | 1742 | 52.8 | 1 | 49 | 1 | 67 | 7 | 0 | 1 | 0 | 1 | 0 | fermented cereals / fermenting plant materials / dairy products / manure / sewage / the faeces and vagina of humans |
| ERR387529 | Lactobacillus frumenti | DSM 13145 | Limosilactobacillus frumenti | type strain | 1730467 | 1676 | 42.6 | 2 | 59 | 0 | 75 | 16 | 0 | 1 | 0 | 1 | 0 | an industrial rye bran fermentation / must / wine / intestine of poultry and swine |
| GCA_001434365.1 | Lactobacillus gastricus | DSM 16045 | Limosilactobacillus gastricus | type strain | 1848461 | 1819 | 41.6 | 3 | 37 | 1 | 81 | 12 | 0 | 0 | 0 | 1 | 0 | biopsy of a human stomach / human milk |
| GCA_001293735.1 | Lactobacillus gorillae | KZ01 | Limosilactobacillus gorillae | type strain | 1641621 | 1568 | 48.1 | 3 | 53 | 1 | 97 |  |  |  |  |  |  | the faeces of a captive gorillas / wild western lowland gorillas |
| ERR387499 | Lactobacillus ingluviei | DSM 15946 | Limosilactobacillus ingluviei | type strain | 2138634 | 2086 | 49.9 | 0 | 66 | 5 | 252 | 4 | 0 | 0 | 0 | 1 | 0 | the crop of a pigeon / birds / cattle / carnivore faeces / Korean rice wine (makgeolii) |
| SRR1151164 | Lactobacillus mucosae | DSM 13345 | Limosilactobacillus mucosae | type strain | 2280266 | 2044 | 46.4 | 0 | 76 | 2 | 163 | 5 | 0 | 1 | 0 | 0 | 1 | the intestine of a pig / the intestine of other vertebrates including humans / type II sourdough / related cereal fermentations |
| GCA_001434465.1 | Lactobacillus oris | DSM 4864 | Limosilactobacillus oris | type strain | 2031774 | 1925 | 50 | 2 | 51 | 2 | 150 | 10 | 0 | 0 | 0 | 1 | 0 | the human saliva / other human body sites including the vagina and mother milk / foods such as corn dough and bran |
| SRR1151250 | Lactobacillus panis | DSM 6035 | Limosilactobacillus panis | type strain | 1986287 | 1887 | 48.1 | 1 | 59 | 2 | 134 | 12 | 0 | 1 | 0 | 0 | 1 | type II sourdough / fermenting plant material / the intestine of birds |
| SRR1151252 | Lactobacillus pontis | DSM 8475 | Limosilactobacillus pontis | type strain | 1656883 | 1614 | 53.5 | 1 | 65 | 0 | 55 | 4 | 1 | 1 | 0 | 1 | 0 | type I and type II sourdough / the intestinal microbiota of swine / silage / dairy products / mezcal fermentation / wet wheat distillers' grain |
| GCA_000010005.1 | Lactobacillus reuteri | JCM 1112 | Limosilactobacillus reuteri | type strain | 2039414 | 2020 | 38.9 | 18 | 65 | 0 | 56 | 9 | 1 | 0 | 0 | 0 | 1 | the intestinal microbiota of rodents, birds, swine, and in other intestinal ecosystems / cereal fermentations / particularly type II sour doughs / Food isolates are of intestinal origin |
| GCA_001437055.1 | Lactobacillus secaliphilus | DSM 17896 | Limosilactobacillus secaliphilus | type strain | 1646143 | 1503 | 47.7 | 2 | 39 | 0 | 99 | 1 | 0 | 0 | 0 | 1 | 0 | type II sourdough |
| SRR1151175 | Lactobacillus vaginalis | DSM 5837 | Limosilactobacillus vaginalis | type strain | 1781526 | 1733 | 40.5 | 0 | 58 | 0 | 67 | 7 | 0 | 1 | 0 | 1 | 0 | microbiota of the human vagina |
| ERR387493 | Lactobacillus aquaticus | DSM 21051 | Liquorilactobacillus aquaticus | type strain | 2399635 | 2210 | 37.4 | 1 | 48 | 1 | 35 | 10 | 1 | 1 | 1 | 0 | 0 | eutrophic freshwater pond |
| SRR1151197 | Lactobacillus cacaonum | DSM 21116 | Liquorilactobacillus cacaonum | type strain | 1917961 | 1823 | 33.9 | 1 | 50 | 1 | 26 | 4 | 1 | 0 | 0 | 1 | 0 | cocoa fermentation |
| ERR387459 | Lactobacillus capillatus | DSM 19910 | Liquorilactobacillus capillatus | type strain | 2224347 | 2107 | 37.6 | 0 | 41 | 1 | 39 | 6 | 1 | 0 | 0 | 1 | 0 | fermented brine used for stinky tofu production |
| SRR1151207 | Lactobacillus ghanensis | DSM 18630 | Liquorilactobacillus ghanensis | type strain | 2602751 | 2416 | 37.1 | 2 | 54 | 1 | 71 | 9 | 0 | 1 | 0 | 1 | 0 | cocoa fermentations |
| ERR387525 | Lactobacillus hordei | DSM 19519 | Liquorilactobacillus hordei | type strain | 2287468 | 2239 | 34.8 | 0 | 57 | 2 | 87 | 8 | 0 | 0 | 0 | 1 | 0 | malted barley / water kefirs / Turkish tradi tional fermented gilaburu fruit juice |
| SRR1151218 | Lactobacillus mali | DSM 20444 | Liquorilactobacillus mali | type strain | 2611318 | 2559 | 36.1 | 2 | 40 | 1 | 94 | 6 | 1 | 0 | 0 | 1 | 0 | wine must / fermenting cider / fermented molasses / water kefirs / cocoa bean fermentations / table olives |
| ERR387505 | Lactobacillus nagelii | DSM 13675 | Liquorilactobacillus nagelii | type strain | 2493596 | 2409 | 36.7 | 0 | 51 | 1 | 98 | 11 | 0 | 1 | 0 | 1 | 0 | partially fermented wine / spontaneous cocoa bean fermentations / water kefirs / fermented cassava food / silage fermentation of fruit residues |
| SRR1151264 | Lactobacillus oeni | DSM 19972 | Liquorilactobacillus oeni | type strain | 2105430 | 1976 | 37.3 | 1 | 52 | 2 | 72 | 4 | 1 | 1 | 1 | 0 | 0 | Bobal wine |
| ERR433495 | Lactobacillus satsumensis | DSM 16230 | Liquorilactobacillus satsumensis | type strain | 2634920 | 2441 | 39.9 | 1 | 48 | 0 | 88 | 5 | 1 | 1 | 0 | 1 | 0 | mashes of shochu / a traditional Japanese distilled spirit made from fermented riceother starchy materials |
| ERR485115 | Lactobacillus sucicola | DSM 21376 | Liquorilactobacillus sucicola | type strain | 2456798 | 2265 | 38.5 | 0 | 55 | 2 | 51 | 8 | 1 | 1 | 0 | 1 | 0 | the sap of an oak (Quercus sp) |
| ERR387550 | Lactobacillus uvarum | DSM 19971 | Liquorilactobacillus uvarum | type strain | 2671380 | 2525 | 36.9 | 1 | 53 | 1 | 94 | 8 | 0 | 0 | 0 | 1 | 0 | Bobal grape musts |
| GCA_000255495.2 | Lactobacillus vini | DSM 20605 | Liquorilactobacillus vini | type strain | 2195706 | 2106 | 37.6 | 3 | 44 | 3 | 58 | 10 | 0 | 1 | 0 | 1 | 0 | fermenting Spanish grape must / bioethanol industrial processes in different distilleries of Brazil |
| SRR1151124 | Lactobacillus bifermentans | DSM 20003 | Loigolactobacillus bifermentans | type strain | 3134903 | 3049 | 44.3 | 3 | 60 | 3 | 225 | 6 | 0 | 0 | 0 | 1 | 0 | spoiled Edam / Gouda cheeses / fermented masau fruits / Himalayan fermented milk products |
| GCA_001433765.1 | Lactobacillus coryniformis subsp. coryniformis | DSM 20001 | Loigolactobacillus coryniformis ssp. coryniformis | type strain | 2705076 | 2579 | 42.9 | 3 | 38 | 1 | 136 | 3 | 1 | 0 |  |  |  | silage / cow dung / dairy barn air and sewage / table olives / wheat / pickled vegetable / cheese and ting / a fermented sorghum porridge |
| SRR1151133 | Lactobacillus coryniformis subsp. torquens | DSM 20004 | Loigolactobacillus coryniformis ssp. torquens | type strain | 2657964 | 2541 | 43 | 2 | 38 | 0 | 132 | 3 | 1 | 0 |  |  |  | cheese / yaks' milk cheese / silage / tomato pomace silage |
| ERR433491 | Lactobacillus rennini | DSM 20253 | Loigolactobacillus rennini | type strain | 2261248 | 2219 | 40.7 | 0 | 54 | 5 | 130 | 10 | 1 | 0 | 0 | 1 | 0 | rennet and are associated with cheese spoilage |
| ERR387532 | Lactobacillus selangorensis | ATCC BAA 66 | Paralactobacillus selangorensis | type strain | 2081509 | 2064 | 46.4 | 1 | 50 | 2 | 125 | 3 | 1 | 0 | 0 | 1 | 0 | a Malaysian food ingredient called chili bo |
| GCA_000829395.1 | Lactobacillus hokkaidonensis | LOOC260 | Paucilactobacillus hokkaidonensis | type strain | 2400586 | 2328 | 38.2 | 12 | 56 | 1 | 36 | 4 | 1 | 0 | 0 | 1 | 0 | grass silage |
| SRR1151187 | Lactobacillus oligofermentans | DSM 15707 | Paucilactobacillus oligofermentans | type strain | 1789353 | 1722 | 35.5 | 2 | 52 | 1 | 17 | 3 | 1 | 0 | 0 | 0 | 1 | marinated poultry meat at the end of its shelf life / fermented olives |
| GCA_001434475.1 | Lactobacillus suebicus | DSM 5007 | Paucilactobacillus suebicus | type strain | 2651315 | 2495 | 39 | 3 | 56 | 2 | 40 | 5 | 1 | 0 |  |  |  | fermented cherry mashes / cider / silage |
| ERR387501 | Lactobacillus vaccinostercus | DSM 20634 | Paucilactobacillus vaccinostercus | type strain | 2553579 | 2440 | 43.5 | 0 | 52 | 0 | 92 | 5 | 0 | 0 |  |  |  | cow dung / fermented tea leaves / fermented cereals |
| GCA_000876205.1 | Lactobacillus wasatchensis | WDC04 | Paucilactobacillus wasatchensis | type strain | 1904253 | 1807 | 39.8 | 3 | 51 | 4 | 22 |  |  |  |  |  |  | spoiled cheddar cheese / silage |
| SRR896433 | Lactobacillus harbinensis | DSM 16991 | Schleiferilactobacillus harbinensis | type strain | 3123257 | 3031 | 53.1 | 1 | 62 | 2 | 228 | 15 | 0 | 0 |  |  |  | fermented vegetables 'Suan Cai' / the brewery environment / / fermented cereals / tomato pomace / spoiled soft drinks |
| SRR1151227 | Lactobacillus perolens | DSM 12744 | Schleiferilactobacillus perolens | type strain | 3269427 | 3106 | 49.2 | 1 | 57 | 2 | 142 | 8 | 0 | 0 |  |  |  | spoiled soft drinks / brewery environments |
| GCA_000469325.1 | Lactobacillus shenzhenensis | LY-73 | Schleiferilactobacillus shenzhenensis | type strain | 3271684 | 2975 | 56.4 | 2 | 43 | 5 | 309 | 16 | 0 | 1 | 0 | 1 | 0 | fermented dairy beverage |
| GCA_001435975.1 | Lactobacillus collinoides | DSM 20515 | Secundilactobacillus collinoides | type strain | 3616190 | 3224 | 46.1 | 3 | 50 | 3 | 241 | 9 | 1 | 0 | 0 | 1 | 0 | compost / apple cider / table olives / dairy products / fermented durian fruit / wines |
| SRR1151214 | Lactobacillus kimchicus | JCM 15530 | Secundilactobacillus kimchicus | type strain | 2593829 | 2511 | 46.6 | 2 | 60 | 4 | 76 | 9 | 1 | 1 | 0 | 1 | 0 | kimchi |
| SRR1151262 | Lactobacillus malefermentans | DSM 5705 | Secundilactobacillus malefermentans | type strain | 2054106 | 2013 | 41 | 3 | 61 | 4 | 70 | 2 | 1 | 0 | 0 | 1 | 0 | beer |
| ERR433478 | Lactobacillus odoratitofui | DSM 19909 | Secundilactobacillus odoratitofui | type strain | 2747284 | 2403 | 44.2 | 1 | 61 | 4 | 79 | 8 | 1 | 0 | 0 | 1 | 0 | fermented brine used for stinky tofu production in Taipei County, Taiwan |
| GCA_000740055.1 | Lactobacillus oryzae | SG293 | Secundilactobacillus oryzae | type strain | 1860394 | 1859 | 42.8 | 6 | 40 | 1 | 96 | 3 | 1 | 1 | 0 | 1 | 0 | fermented rice grains in Tochigi, Japan |
| SRR1151134 | Lactobacillus paracollinoides | DSM 15502 | Secundilactobacillus paracollinoides | type strain | 3470681 | 3214 | 46.9 | 2 | 67 | 2 | 288 | 4 | 1 | 0 | 0 | 1 | 0 | beer / cider / fermented olives |
| GCA_001313225.1 | Lactobacillus silagei | JCM 19001 | Secundilactobacillus silagei | type strain | 2650200 | 3600 | 44.8 | 3 | 61 | 4 | 243 |  |  |  |  |  |  | silage |
| ERR387542 | Lactobacillus similis | DSM 23365 | Secundilactobacillus similis | type strain | 3452668 | 3084 | 47 | 0 | 49 | 3 | 219 | 8 | 1 | 0 | 0 | 1 | 0 | fermented cane molasses at alcohol plants in Thailand / rice wine (makgeolii) |

Table S1. Features of the 178 LAB strains. The accession numbers of the genome sequences, the old and new species names, strain names, type status, seven genomic features, six phenotypic characteristics, and the strains’ isolation source are presented. The genomic features are genome size (bp), number of CDS, G/C content (%), number of rRNA, number of tRNA, number of CRISPRs, number of CDS judged to be HGTs. One of phenotypes is sugar utilization value which indicates the number of sugar types that can be utilized. The other five phenotypes, growth at 15 °C, growth at 45 °C, and growth in microaerobic, facultatively anaerobic, and obligate anaerobic conditions were expressed as a dummy variable: If a strain has the feature, 1 was given as the dummy variable and 0 if not. The isolation source indicates the environment in which the species was isolated.
