# Supplementary material for: A sugar utilization phenotype contributes to the formation of genetic exchange communities in lactic acid bacteria
Source: FEMS Microbiol Lett. 2021 Sep 1;368(17):fnab117. doi: 10.1093/femsle/fnab117 (PMC8440127; doi:10.1093/femsle/fnab117)
Supplement: fnab117_Supplemental_Files [file fnab117_supplemental_files.zip › Supplementary_data_Table_S2.docx]

| community | number of strains | genus | member |
| --- | --- | --- | --- |
| 1 | 7 | Lactiplantibacillus | Lactiplantibacillus_fabifermentans_DSM_21115,Lactiplantibacillus_xiangfangensis_LMG_26013,Lactiplantibacillus_paraplantarum_DSM_10667,Lactiplantibacillus_herbarum_TCF032-E4,Lactiplantibacillus_plantarum_ssp._plantarum_CGMCC_1.2437,Lactiplantibacillus_pentosus_DSM_20314,Lactiplantibacillus_plantarum_ssp._argentoratensis_DSM_16365 |
| 2 | 3 | Lactiplantibacillus,Loigolactobacillus | Loigolactobacillus__bifermentans_DSM_20003,Lactiplantibacillus_pentosus_DSM_20314,Lactiplantibacillus_plantarum_ssp._argentoratensis_DSM_16365 |
| 3 | 4 | Lactiplantibacillus,Levilactobacillus | Levilactobacillus_acidifarinae_DSM_19394,Lactiplantibacillus_paraplantarum_DSM_10667,Lactiplantibacillus_plantarum_ssp._plantarum_CGMCC_1.2437,Lactiplantibacillus_plantarum_ssp._argentoratensis_DSM_16365 |
| 4 | 2 | Limosilactobacillus | Limosilactobacillus_frumenti_DSM_13145,Limosilactobacillus_vaginalis_DSM_5837 |
| 5 | 2 | Limosilactobacillus | Limosilactobacillus_frumenti_DSM_13145,Limosilactobacillus_panis_DSM_6035 |
| 6 | 7 | Schleiferilactobacillus,Lacticaseibacillus,Lactiplantibacillus | Schleiferilactobacillus_harbinensis_DSM_16991,Lactiplantibacillus_paraplantarum_DSM_10667,Lacticaseibacillus_rhamnosus_DSM_20021,Lacticaseibacillus_casei_DSM_20178,Lacticaseibacillus_casei_ATCC_393,Schleiferilactobacillus_perolens_DSM_12744,Lacticaseibacillus_saniviri_DSM_24301 |
| 7 | 7 | Schleiferilactobacillus,Lacticaseibacillus,Agrilactobacillus,Lactiplantibacillus | Schleiferilactobacillus_harbinensis_DSM_16991,Lactiplantibacillus_paraplantarum_DSM_10667,Lacticaseibacillus_rhamnosus_DSM_20021,Lacticaseibacillus_casei_DSM_20178,Lacticaseibacillus_casei_ATCC_393,Schleiferilactobacillus_perolens_DSM_12744,Agrilactobacillus_composti_DSM_18527 |
| 8 | 7 | Schleiferilactobacillus,Lacticaseibacillus,Lactiplantibacillus | Schleiferilactobacillus_harbinensis_DSM_16991,Lactiplantibacillus_paraplantarum_DSM_10667,Lacticaseibacillus_rhamnosus_DSM_20021,Lacticaseibacillus_casei_DSM_20178,Lacticaseibacillus_casei_ATCC_393,Lactiplantibacillus_pentosus_DSM_20314,Lacticaseibacillus_saniviri_DSM_24301 |
| 9 | 7 | Schleiferilactobacillus,Lacticaseibacillus,Agrilactobacillus,Lactiplantibacillus | Schleiferilactobacillus_harbinensis_DSM_16991,Lactiplantibacillus_paraplantarum_DSM_10667,Lacticaseibacillus_rhamnosus_DSM_20021,Lacticaseibacillus_casei_DSM_20178,Lacticaseibacillus_casei_ATCC_393,Lactiplantibacillus_pentosus_DSM_20314,Agrilactobacillus_composti_DSM_18527 |
| 10 | 7 | Schleiferilactobacillus,Lacticaseibacillus,Lactiplantibacillus | Schleiferilactobacillus_harbinensis_DSM_16991,Lactiplantibacillus_paraplantarum_DSM_10667,Lacticaseibacillus_rhamnosus_DSM_20021,Lacticaseibacillus_casei_DSM_20178,Lactiplantibacillus_plantarum_ssp._plantarum_CGMCC_1.2437,Schleiferilactobacillus_perolens_DSM_12744,Lacticaseibacillus_saniviri_DSM_24301 |
| 11 | 7 | Schleiferilactobacillus,Lacticaseibacillus,Agrilactobacillus,Lactiplantibacillus | Schleiferilactobacillus_harbinensis_DSM_16991,Lactiplantibacillus_paraplantarum_DSM_10667,Lacticaseibacillus_rhamnosus_DSM_20021,Lacticaseibacillus_casei_DSM_20178,Lactiplantibacillus_plantarum_ssp._plantarum_CGMCC_1.2437,Schleiferilactobacillus_perolens_DSM_12744,Agrilactobacillus_composti_DSM_18527 |
| 12 | 7 | Schleiferilactobacillus,Lacticaseibacillus,Lactiplantibacillus | Schleiferilactobacillus_harbinensis_DSM_16991,Lactiplantibacillus_paraplantarum_DSM_10667,Lacticaseibacillus_rhamnosus_DSM_20021,Lacticaseibacillus_casei_DSM_20178,Lactiplantibacillus_plantarum_ssp._plantarum_CGMCC_1.2437,Lactiplantibacillus_pentosus_DSM_20314,Lacticaseibacillus_saniviri_DSM_24301 |
| 13 | 7 | Schleiferilactobacillus,Lacticaseibacillus,Agrilactobacillus,Lactiplantibacillus | Schleiferilactobacillus_harbinensis_DSM_16991,Lactiplantibacillus_paraplantarum_DSM_10667,Lacticaseibacillus_rhamnosus_DSM_20021,Lacticaseibacillus_casei_DSM_20178,Lactiplantibacillus_plantarum_ssp._plantarum_CGMCC_1.2437,Lactiplantibacillus_pentosus_DSM_20314,Agrilactobacillus_composti_DSM_18527 |
| 14 | 7 | Schleiferilactobacillus,Lactiplantibacillus,Latilactobacillus,Lacticaseibacillus,Paucilactobacillus | Schleiferilactobacillus_harbinensis_DSM_16991,Latilactobacillus_sakei_ssp._carnosus_DSM_15831,Lacticaseibacillus_casei_ATCC_393,Lacticaseibacillus_casei_DSM_20178,Lacticaseibacillus_saniviri_DSM_24301,Lactiplantibacillus_pentosus_DSM_20314,Paucilactobacillus_hokkaidonensis_LOOC260 |
| 15 | 4 | Schleiferilactobacillus,Lacticaseibacillus | Schleiferilactobacillus_harbinensis_DSM_16991,Schleiferilactobacillus_shenzhenensis_LY-73,Lacticaseibacillus_saniviri_DSM_24301,Lacticaseibacillus_brantae_DSM_23927 |
| 16 | 7 | Schleiferilactobacillus,Lacticaseibacillus | Schleiferilactobacillus_harbinensis_DSM_16991,Schleiferilactobacillus_shenzhenensis_LY-73,Lacticaseibacillus_saniviri_DSM_24301,Lacticaseibacillus_rhamnosus_DSM_20021,Lacticaseibacillus_casei_DSM_20178,Lacticaseibacillus_casei_ATCC_393,Schleiferilactobacillus_perolens_DSM_12744 |
| 17 | 7 | Schleiferilactobacillus,Lacticaseibacillus,Lactiplantibacillus | Schleiferilactobacillus_harbinensis_DSM_16991,Schleiferilactobacillus_shenzhenensis_LY-73,Lacticaseibacillus_saniviri_DSM_24301,Lacticaseibacillus_rhamnosus_DSM_20021,Lacticaseibacillus_casei_DSM_20178,Lacticaseibacillus_casei_ATCC_393,Lactiplantibacillus_pentosus_DSM_20314 |
| 18 | 7 | Schleiferilactobacillus,Lacticaseibacillus,Lactiplantibacillus | Schleiferilactobacillus_harbinensis_DSM_16991,Schleiferilactobacillus_shenzhenensis_LY-73,Lacticaseibacillus_saniviri_DSM_24301,Lacticaseibacillus_rhamnosus_DSM_20021,Lacticaseibacillus_casei_DSM_20178,Lactiplantibacillus_plantarum_ssp._plantarum_CGMCC_1.2437,Schleiferilactobacillus_perolens_DSM_12744 |
| 19 | 7 | Schleiferilactobacillus,Lacticaseibacillus,Lactiplantibacillus | Schleiferilactobacillus_harbinensis_DSM_16991,Schleiferilactobacillus_shenzhenensis_LY-73,Lacticaseibacillus_saniviri_DSM_24301,Lacticaseibacillus_rhamnosus_DSM_20021,Lacticaseibacillus_casei_DSM_20178,Lactiplantibacillus_plantarum_ssp._plantarum_CGMCC_1.2437,Lactiplantibacillus_pentosus_DSM_20314 |
| 20 | 5 | Schleiferilactobacillus,Lacticaseibacillus | Schleiferilactobacillus_harbinensis_DSM_16991,Schleiferilactobacillus_shenzhenensis_LY-73,Lacticaseibacillus_paracasei_ssp._paracasei_JCM_8130,Lacticaseibacillus_sharpeae_DSM_20505,Schleiferilactobacillus_perolens_DSM_12744 |
| 21 | 5 | Schleiferilactobacillus,Lacticaseibacillus | Schleiferilactobacillus_harbinensis_DSM_16991,Schleiferilactobacillus_shenzhenensis_LY-73,Lacticaseibacillus_paracasei_ssp._paracasei_JCM_8130,Lacticaseibacillus_sharpeae_DSM_20505,Lacticaseibacillus_manihotivorans_DSM_13343 |
| 22 | 9 | Schleiferilactobacillus,Lacticaseibacillus | Schleiferilactobacillus_harbinensis_DSM_16991,Schleiferilactobacillus_shenzhenensis_LY-73,Lacticaseibacillus_paracasei_ssp._paracasei_JCM_8130,Lacticaseibacillus_rhamnosus_DSM_20021,Lacticaseibacillus_casei_DSM_20178,Lacticaseibacillus_casei_ATCC_393,Lacticaseibacillus_paracasei_ATCC_334,Schleiferilactobacillus_perolens_DSM_12744,Lacticaseibacillus_paracasei_ssp._tolerans_DSM_20258 |
| 23 | 9 | Schleiferilactobacillus,Lacticaseibacillus,Agrilactobacillus | Schleiferilactobacillus_harbinensis_DSM_16991,Schleiferilactobacillus_shenzhenensis_LY-73,Lacticaseibacillus_paracasei_ssp._paracasei_JCM_8130,Lacticaseibacillus_rhamnosus_DSM_20021,Lacticaseibacillus_casei_DSM_20178,Lacticaseibacillus_casei_ATCC_393,Lacticaseibacillus_paracasei_ATCC_334,Schleiferilactobacillus_perolens_DSM_12744,Agrilactobacillus_composti_DSM_18527 |
| 24 | 9 | Schleiferilactobacillus,Lacticaseibacillus | Schleiferilactobacillus_harbinensis_DSM_16991,Schleiferilactobacillus_shenzhenensis_LY-73,Lacticaseibacillus_paracasei_ssp._paracasei_JCM_8130,Lacticaseibacillus_rhamnosus_DSM_20021,Lacticaseibacillus_casei_DSM_20178,Lacticaseibacillus_casei_ATCC_393,Lacticaseibacillus_paracasei_ATCC_334,Schleiferilactobacillus_perolens_DSM_12744,Lacticaseibacillus_camelliae_DSM_22697 |
| 25 | 9 | Schleiferilactobacillus,Lacticaseibacillus | Schleiferilactobacillus_harbinensis_DSM_16991,Schleiferilactobacillus_shenzhenensis_LY-73,Lacticaseibacillus_paracasei_ssp._paracasei_JCM_8130,Lacticaseibacillus_rhamnosus_DSM_20021,Lacticaseibacillus_casei_DSM_20178,Lacticaseibacillus_casei_ATCC_393,Lacticaseibacillus_paracasei_ATCC_334,Lacticaseibacillus_manihotivorans_DSM_13343,Lacticaseibacillus_camelliae_DSM_22697 |
| 26 | 8 | Schleiferilactobacillus,Lacticaseibacillus,Agrilactobacillus,Lactiplantibacillus | Schleiferilactobacillus_harbinensis_DSM_16991,Schleiferilactobacillus_shenzhenensis_LY-73,Lacticaseibacillus_paracasei_ssp._paracasei_JCM_8130,Lacticaseibacillus_rhamnosus_DSM_20021,Lacticaseibacillus_casei_DSM_20178,Lacticaseibacillus_casei_ATCC_393,Lactiplantibacillus_pentosus_DSM_20314,Agrilactobacillus_composti_DSM_18527 |
| 27 | 8 | Schleiferilactobacillus,Lacticaseibacillus,Agrilactobacillus,Lactiplantibacillus | Schleiferilactobacillus_harbinensis_DSM_16991,Schleiferilactobacillus_shenzhenensis_LY-73,Lacticaseibacillus_paracasei_ssp._paracasei_JCM_8130,Lacticaseibacillus_rhamnosus_DSM_20021,Lacticaseibacillus_casei_DSM_20178,Lactiplantibacillus_plantarum_ssp._plantarum_CGMCC_1.2437,Agrilactobacillus_composti_DSM_18527,Schleiferilactobacillus_perolens_DSM_12744 |
| 28 | 8 | Schleiferilactobacillus,Lacticaseibacillus,Agrilactobacillus,Lactiplantibacillus | Schleiferilactobacillus_harbinensis_DSM_16991,Schleiferilactobacillus_shenzhenensis_LY-73,Lacticaseibacillus_paracasei_ssp._paracasei_JCM_8130,Lacticaseibacillus_rhamnosus_DSM_20021,Lacticaseibacillus_casei_DSM_20178,Lactiplantibacillus_plantarum_ssp._plantarum_CGMCC_1.2437,Agrilactobacillus_composti_DSM_18527,Lactiplantibacillus_pentosus_DSM_20314 |
| 29 | 3 | Schleiferilactobacillus,Lapidilactobacillus | Schleiferilactobacillus_harbinensis_DSM_16991,Schleiferilactobacillus_shenzhenensis_LY-73,Lapidilactobacillus_concavus_DSM_17758 |
| 30 | 3 | Lacticaseibacillus,Lactiplantibacillus,Secundilactobacillus | Secundilactobacillus_kimchicus_JCM_15530,Lacticaseibacillus_casei_DSM_20178,Lactiplantibacillus_plantarum_ssp._plantarum_CGMCC_1.2437 |
| 31 | 2 | Schleiferilactobacillus,Lactobacillus | Lactobacillus_melliventris_Hma8,Schleiferilactobacillus_perolens_DSM_12744 |
| 32 | 2 | Liquorilactobacillus | Liquorilactobacillus_nagelii_DSM_13675,Liquorilactobacillus_ghanensis_DSM_18630 |
| 33 | 3 | Lactiplantibacillus,Liquorilactobacillus | Liquorilactobacillus_nagelii_DSM_13675,Lactiplantibacillus_paraplantarum_DSM_10667,Lactiplantibacillus_plantarum_ssp._plantarum_CGMCC_1.2437 |
| 34 | 6 | Lactiplantibacillus,Agrilactobacillus | Lactiplantibacillus_xiangfangensis_LMG_26013,Lactiplantibacillus_paraplantarum_DSM_10667,Lactiplantibacillus_plantarum_ssp._plantarum_CGMCC_1.2437,Agrilactobacillus_composti_DSM_18527,Lactiplantibacillus_pentosus_DSM_20314,Lactiplantibacillus_plantarum_ssp._argentoratensis_DSM_16365 |
| 35 | 8 | Lactiplantibacillus,Lacticaseibacillus,Agrilactobacillus,Schleiferilactobacillus | Lactiplantibacillus_plantarum_ssp._argentoratensis_DSM_16365,Lacticaseibacillus_rhamnosus_DSM_20021,Agrilactobacillus_composti_DSM_18527,Lacticaseibacillus_casei_DSM_20178,Lacticaseibacillus_casei_ATCC_393,Lacticaseibacillus_paracasei_ssp._paracasei_JCM_8130,Lacticaseibacillus_paracasei_ATCC_334,Schleiferilactobacillus_perolens_DSM_12744 |
| 36 | 7 | Lactiplantibacillus,Lacticaseibacillus,Agrilactobacillus | Lactiplantibacillus_plantarum_ssp._argentoratensis_DSM_16365,Lacticaseibacillus_rhamnosus_DSM_20021,Agrilactobacillus_composti_DSM_18527,Lacticaseibacillus_casei_DSM_20178,Lacticaseibacillus_casei_ATCC_393,Lacticaseibacillus_paracasei_ssp._paracasei_JCM_8130,Lactiplantibacillus_pentosus_DSM_20314 |
| 37 | 7 | Lactiplantibacillus,Lacticaseibacillus,Agrilactobacillus,Schleiferilactobacillus | Lactiplantibacillus_plantarum_ssp._argentoratensis_DSM_16365,Lacticaseibacillus_rhamnosus_DSM_20021,Agrilactobacillus_composti_DSM_18527,Lacticaseibacillus_casei_DSM_20178,Lacticaseibacillus_casei_ATCC_393,Lactiplantibacillus_paraplantarum_DSM_10667,Schleiferilactobacillus_perolens_DSM_12744 |
| 38 | 7 | Lactiplantibacillus,Lacticaseibacillus,Agrilactobacillus | Lactiplantibacillus_plantarum_ssp._argentoratensis_DSM_16365,Lacticaseibacillus_rhamnosus_DSM_20021,Agrilactobacillus_composti_DSM_18527,Lacticaseibacillus_casei_DSM_20178,Lacticaseibacillus_casei_ATCC_393,Lactiplantibacillus_paraplantarum_DSM_10667,Lactiplantibacillus_pentosus_DSM_20314 |
| 39 | 7 | Lactiplantibacillus,Lacticaseibacillus,Agrilactobacillus,Schleiferilactobacillus | Lactiplantibacillus_plantarum_ssp._argentoratensis_DSM_16365,Lacticaseibacillus_rhamnosus_DSM_20021,Agrilactobacillus_composti_DSM_18527,Lacticaseibacillus_casei_DSM_20178,Lactiplantibacillus_plantarum_ssp._plantarum_CGMCC_1.2437,Schleiferilactobacillus_perolens_DSM_12744,Lacticaseibacillus_paracasei_ssp._paracasei_JCM_8130 |
| 40 | 7 | Lactiplantibacillus,Lacticaseibacillus,Agrilactobacillus,Schleiferilactobacillus | Lactiplantibacillus_plantarum_ssp._argentoratensis_DSM_16365,Lacticaseibacillus_rhamnosus_DSM_20021,Agrilactobacillus_composti_DSM_18527,Lacticaseibacillus_casei_DSM_20178,Lactiplantibacillus_plantarum_ssp._plantarum_CGMCC_1.2437,Schleiferilactobacillus_perolens_DSM_12744,Lactiplantibacillus_paraplantarum_DSM_10667 |
| 41 | 7 | Lactiplantibacillus,Lacticaseibacillus,Agrilactobacillus | Lactiplantibacillus_plantarum_ssp._argentoratensis_DSM_16365,Lacticaseibacillus_rhamnosus_DSM_20021,Agrilactobacillus_composti_DSM_18527,Lacticaseibacillus_casei_DSM_20178,Lactiplantibacillus_plantarum_ssp._plantarum_CGMCC_1.2437,Lactiplantibacillus_pentosus_DSM_20314,Lacticaseibacillus_paracasei_ssp._paracasei_JCM_8130 |
| 42 | 7 | Lactiplantibacillus,Lacticaseibacillus,Agrilactobacillus | Lactiplantibacillus_plantarum_ssp._argentoratensis_DSM_16365,Lacticaseibacillus_rhamnosus_DSM_20021,Agrilactobacillus_composti_DSM_18527,Lacticaseibacillus_casei_DSM_20178,Lactiplantibacillus_plantarum_ssp._plantarum_CGMCC_1.2437,Lactiplantibacillus_pentosus_DSM_20314,Lactiplantibacillus_paraplantarum_DSM_10667 |
| 43 | 2 | Lactiplantibacillus,Liquorilactobacillus | Lactiplantibacillus_plantarum_ssp._argentoratensis_DSM_16365,Liquorilactobacillus_sucicola_DSM_21376 |
| 44 | 3 | Lactiplantibacillus,Latilactobacillus | Lactiplantibacillus_plantarum_ssp._argentoratensis_DSM_16365,Latilactobacillus_fuchuensis_JCM_11249,Lactiplantibacillus_plantarum_ssp._plantarum_CGMCC_1.2437 |
| 45 | 2 | Companilactobacillus | Companilactobacillus_kimchiensis_DSM_24716,Companilactobacillus_nantensis_DSM_16982 |
| 46 | 2 | Companilactobacillus | Companilactobacillus_ginsenosidimutans_EMML_3141,Companilactobacillus_nantensis_DSM_16982 |
| 47 | 2 | Lactiplantibacillus,Secundilactobacillus | Secundilactobacillus_similis_DSM_23365,Lactiplantibacillus_pentosus_DSM_20314 |
| 48 | 2 | Lactiplantibacillus,Liquorilactobacillus | Liquorilactobacillus_uvarum_DSM_19971,Lactiplantibacillus_paraplantarum_DSM_10667 |
| 49 | 2 | Ligilactobacillus | Ligilactobacillus_agilis_DSM_20509,Ligilactobacillus_ruminis_ATCC_27780 |
| 50 | 2 | Lactiplantibacillus,Loigolactobacillus | Loigolactobacillus_rennini_DSM_20253,Lactiplantibacillus_pentosus_DSM_20314 |
| 51 | 2 | Lacticaseibacillus,Companilactobacillus | Companilactobacillus_nantensis_DSM_16982,Lacticaseibacillus_casei_DSM_20178 |

Table S2. Community extraction of the networks of the shared generalist group orthologs. The table indicates the number of strains, genera name, and member in each community for the generalist group ortholog networks.
