# Supplementary material for: A sugar utilization phenotype contributes to the formation of genetic exchange communities in lactic acid bacteria
Source: FEMS Microbiol Lett. 2021 Sep 1;368(17):fnab117. doi: 10.1093/femsle/fnab117 (PMC8440127; doi:10.1093/femsle/fnab117)
Supplement: fnab117_Supplemental_Files [file fnab117_supplemental_files.zip › Supplementary_data_Table_S3.docx]

| group | product |
| --- | --- |
| generalist group ortholog | hypothetical protein |
|  | bacteriocin immunity protein |
|  | prophage protein |
|  | XRE family transcriptional regulator |
|  | membrane protein |
|  | adenylyl transferase |
|  | nitroreductase |
|  | maltodextrose utilization protein malA |
|  | integral membrane protein PlnU |
|  | peptidase S41 |
|  | beta-lactamase |
|  | L-lactate dehydrogenase |
|  | mucus-binding protein |
|  | DNA mismatch repair protein MutS |
|  | polysaccharide lyase family 8 |
|  | RNA polymerase sigma factor SigV |
|  | MerR family transcriptional regulator |
|  | alcohol dehydrogenase |
|  | 4-hydroxyphenylacetate-3-hydroxylase |
|  | ABC transporter permease protein |
|  | L-fucose isomerase |
|  | major head protein Cps |
|  | phage portal protein |
|  | lipoprotein LipO precursor |
|  | spermidine/putrescine ABC transporter permease protein |
|  | GNAT family acetyltransferase |
|  | MarR family transcriptional regulator |
|  | RNA-binding protein |
|  | two-component system response regulator |
|  | LysR family transcriptional regulator |
|  | exopolysaccharide biosynthesis protein |
|  | TetR family transcriptional regulator |
|  | iron ABC transporter permease protein |
|  | MutR family transcriptional regulator |
|  | cupin |
|  | aspartate aminotransferase |
|  | adherence-associated mucus-binding protein,LPXTG-motif cell wall anchor |
|  | extracellular zinc metalloproteinase |
|  | ribitolphosphotransferase |
|  | sulfate adenylyltransferase |
|  | adenylylsulfate kinase |
|  | AraC family transcriptional regulator |
|  | major facilitator superfamily transporter |
|  | ArsR family transcriptional regulator |
|  | extracellular lipoprotein precursor, Asp-rich |
|  | mannosyl-glycoprotein endo-beta-N-acetylglucosaminidase |
|  | DeoR family transcriptional regulator |
|  | BglG family transcriptional antiterminator/PTS system mannitol/fructose-specific IIA component |
|  | cell surface hydrolase |
|  | NUDIX family hydrolase |
|  | lipoprotein |
|  | PadR family transcriptional regulator |
|  | phage envelope protein |
|  | Cro/Cl family transcriptional regulator |
|  | NADPH-dependent FMN reductase family protein |
|  | universal stress protein UspA |
|  | tail fiber |
|  | 3-dehydroquinate dehydratase |
|  | integral membrane protein |
|  | phosphoglycerate mutase |
|  | extracellular protein |
|  | conjugal transfer protein |
|  | capsular polysaccharide biosynthesis protein |
|  | beta-glucosides-specific PTS system IIC component |
|  | beta-glucosides-specific PTS system IIB component |
|  | hemagglutinin |
|  | DNA-3-methyladenine glycosylase I |
|  | ribonuclease HI |
|  | glycosyl transferase family 1 |
|  | gp1 protein |
|  | phage major tail protein |
|  | mannose/fructose/sorbose-specific PTS system IIA component |
|  | preprotein translocase subunit YajC |
|  | cytochrome d ubiquinol oxidase subunit II |
|  | acetyltransferase |
|  | DNA-binding protein with HIRAN domain protein |
|  | sodium/sulfate symport protein |
|  | prebacteriocin |
|  | bacteriocin immunity protein PlnL |
|  | MATE family efflux transporter |
|  | PTS sugar transporter IIA component |
|  | serine transporter |
|  | frv operon regulatory protein |
|  | phage related protein |
|  | sugar ABC transporter permease protein |
|  | cell surface protein |
|  | phage protein |
|  | fibrinogen-binding protein |
|  | transposase |
|  | 3',5'-cyclic-nucleotide phosphodiesterase |
|  | D-galactose-binding periplasmic protein precursor |
|  | 16S rRNA methyltransferase |
|  | ATPase component of ABC transporter with duplicated ATPase domains |
|  | LuxR family transcriptional regulator |
|  | short-chain dehydrogenase/oxidoreductase |
|  | iron ABC transporter substrate-binding protein |
|  | sodium:proton antiporter |
|  | glycosyl transferase |
|  | polysaccharide biosynthesis protein |
|  | transcriptional regulator |
|  | GntR family transcriptional regulator |
|  | glycerophosphoryl diester phosphodiesterase family protein |
|  | oligoendopeptidase F |
|  | sugar ABC transporter substrate-binding protein |
|  | mannose-specific adhesin, LPXTG-motif cell wall anchor |
|  | ABC-2 transporter family protein |
|  | beta-lactamase family protein |
|  | competence protein TfoX |
|  | sensory box protein/response regulator |
|  | sortase |
|  | putative membrane protein |
|  | 5-methyltetrahydropteroyltriglutamate-- homocysteine methyltransferase |
|  | BetT protein |
|  | polysaccharide polymerase |
|  | integral membrane protein (putative) |
|  | potassium transporter Kup |
|  | chitin-binding protein |
|  | phosphohydrolase |
|  | PTS sugar transporter subunit IIA |
|  | putative chromate transport protein |
|  | group II intron reverse transcriptase/maturase |
|  | transcriptional antiterminator |
|  | peptidase family S41 |
|  | helix-turn-helix protein |
|  | cellulase (glycosyl hydrolase family 5) |
|  | alpha-L-fucosidase |
|  | deoxyuridine 5'-triphosphate nucleotidohydrolase |
|  | cytosolic protein |
|  | mannose/fructose/N-acetylgalactosamine-specific PTS system transporter subunit IID |
|  | putative secreted protein |
|  | major Facilitator Superfamily protein |
|  | pyruvate kinase |
|  | NAD/NADP octopine/nopaline dehydrogenase |
|  | putative sporulation-specific glycosylase YdhD |
|  | peptide methionine sulfoxide reductase |
|  | methyl-accepting chemotaxis sensory transducer |
|  | FIST N domain protein |
|  | ankyrin repeat family protein |
|  | sigma-70, region 4 |
|  | transcriptional regulator/sugar kinase NagC |
|  | alpha/beta hydrolase |
|  | flippase |
|  | S-adenosyl-L-homocysteine hydrolase, NAD binding domain protein |
|  | WaaG-like sugar transferase |
|  | ABC-2 family transporter protein |
|  | minor capsid protein |
|  | cellobiose-specific PTS system IIC component |
|  | cell surface protein, CscB family |
|  | branched-chain amino acid ABC transporter ATP-binding protein |
|  | alpha-amylase |
|  | UTP--glucose-1-phosphate uridylyltransferase |
|  | thioredoxin domain protein |
|  | CDP-diacylglycerol--glycerol-3-phosphate 3-phosphatidyltransferase |
|  | transglutaminase-like superfamily protein |
|  | serine protease |
|  | SpoVT / AbrB like domain protein |
|  | phosphate ABC transporter substrate-binding protein |
|  | YhhN-like protein |
|  | phosphoketolase |
|  | rhomboid family protein |
|  | poly(glycerol-phosphate) alpha-glucosyltransferase |
|  | acetate kinase |
|  | molecular chaperone DnaK |
|  | ABC transporter substrate-binding protein |
|  | Heparinase II/III-like protein |
|  | single-stranded DNA-binding protein |
|  | acetyltransferase (GNAT) family protein |
|  | alpha-galactosidase |
|  | chromosome partition protein Smc |
|  | Na+/xyloside symporter related transporter |
|  | AbrB family transcriptional regulator |
|  | DNA-binding protein |
|  | LPXTG-motif cell wall anchor domain protein |
|  | putative signal transduction protein with a C-terminal HATPase domain protein |
|  | sugar O-acyltransferase |
|  | plantaricin biosynthesis protein PlnR |
|  | haloacid dehalogenase |
|  | surface antigen |
|  | acyltransferase family protein |
|  | HTH-type transcriptional regulator MhqR |
|  | type 1 restriction modification systemspecificity protein |
|  | tellurite resistance protein TerB |
|  | chromosome partitioning protein ParA |
|  | thymidylate kinase |
|  | holin |
|  | tail protein |
|  | accessory gene regulator AgrB |
|  | NADPH:quinone reductase |
|  | antimicrobial peptide ABC transporter ATP-binding protein |
|  | putative recombinase |
|  | L-serine dehydratase beta subunit |
|  | glycerol-3-phosphate dehydrogenase |
|  | Fe-S-cluster oxidoreductase |
|  | N-acetyltransferase |
|  | Pectate lyase precursor |
|  | multidrug ABC transporter ATP-binding and permease protein |
|  | hydrolase |
|  | glycosyl transferase family 2 |
|  | tryptophan synthase alpha chain |
|  | ascorbate-specific PTS system IIC component |
|  | iron-sulfur cluster binding protein/lactate utilization protein LutB |
|  | ABC transporter ATP-binding component |
|  | Mob |
|  | amino acid permease |
|  | diacylglyceryl transferase |
|  | beta-galactosidase |
|  | SnoaL-like polyketide cyclase |
|  | phage holin protein (Holin_LLH) |
|  | muramidase |
|  | potassium transporter TrkA |
|  | two-component system sensor histidine kinase |
|  | putative nucleotidyltransferase |
|  | Assimilatory nitrite reductase [NAD(P)H] small subunit |
|  | peptidoglycan-binding protein |
|  | butyrate-acetoacetate CoA-transferase, beta subunit |
|  | Fe-S oxidoreductase |
|  | dipeptide/tripeptide permease |
|  | transcription regulator |
|  | RNA polymerase sigma factor |
|  | penicillin-binding protein 2B |
|  | acetylornithine deacetylase/succinyl-diaminopimelate desuccinylase |
|  | sugar O-acetyltransferase |
|  | short chain dehydrogenase |
|  | cellobiose-specific PTS system IIB component |
|  | phage single-strand DNA binding protein |
|  | ATP-dependent nuclease, subunit B |
|  | ABC transporter ATP-binding protein |
|  | mannose/fructose/sorbose-specific PTS system IID component |
|  | phospholipase/Carboxylesterase |
|  | cell division protein FtsK |
|  | alpha-glucosidase |
|  | glycoside hydrolase |
|  | plantaricin biosynthesis protein PlnQ |
|  | plantaricin A precursor peptide, induction factor |
|  | bacteriocin precursor peptide PlnF |
|  | bacteriocin precursor peptide PlnE |
|  | peptidylprolyl isomerase |
|  | proton glutamate symport protein |
|  | minor capsid protein from bacteriophage |
|  | flagellar biosynthetic protein FlhB |
|  | RNHCP domain protein |
|  | serine/threonine-protein kinase PknD |
|  | Capsid protein (F protein) |
|  | Microvirus J protein |
|  | bacteriophage scaffolding protein D |
|  | phage protein C |
|  | bacteriophage replication gene A protein (GPA) |
|  | Microvirus H protein (pilot protein) |
| specialist group ortholog | hypothetical protein |
|  | membrane protein |
|  | signal transduction diguanylate cyclase |
|  | transposase |
|  | competence protein ComGF |
|  | transcriptional regulator |
|  | acetyltransferase |
|  | dipeptidase |
|  | oligopeptide ABC transporter substrate-binding protein |
|  | HTH-type transcriptional regulator Hpr |
|  | permease |
|  | glycogen phosphorylase |
|  | proline dipeptidase |
|  | antimicrobial peptide ABC transporter ATP-binding protein |
|  | transcriptional regulator/sugar kinase NagC |
|  | MATE efflux family protein |
|  | extracellular zinc metalloproteinase |
|  | phosphoenolpyruvate carboxykinase |
|  | ATP-dependent DNA helicase RecQ |
|  | serine hydroxymethyltransferase |
|  | small membrane protein |
|  | phosphopentomutase |
|  | type III restriction enzyme, res subunit |
|  | putative helicase |
|  | NgoFVII restriction endonuclease |
|  | N-acetyltransferase |
|  | glycerol uptake facilitator protein |
|  | ABC transporter permease protein |
|  | D-lactate dehydrogenase |
|  | multidrug ABC transporter ATP-binding and permease protein |
|  | esterase |
|  | Xaa-Pro aminopeptidase |
|  | glycerol kinase |
|  | fumarate reductase |
|  | fumarate reductase flavoprotein subunit |
|  | branched-chain amino acid permease |
|  | ABC transporter ATP-binding protein |
|  | drug/metabolite transporter permease |
|  | septum formation initiation protein |
|  | dihydroorotate dehydrogenase |
|  | 2-dehydropantoate 2-reductase |
|  | zinc ABC transporter substrate-binding protein |
|  | putative deoxyribodipyrimidine photolyase |
|  | 2', 3'-cyclic nucleotide 2'-phosphodiesterase |
|  | beta-lactamase class A |
|  | surface protein Rib |
|  | beta-galactosidase |
|  | cell division protein |
|  | Nuclease-related domain protein |
|  | DNA damage-indicible protein DnaD |
|  | Na+/H+ antiporter |
|  | TM2 domain protein |
|  | processive diacylglycerol beta-glucosyltransferase |
|  | alpha/beta hydrolase family protein |
|  | GntR family transcriptional regulator |
|  | aminotransferase |
|  | acyltransferase |
|  | asparagine synthase |
|  | sugar O-acetyltransferase |
|  | DeoR family transcriptional regulator |
|  | alpha-amylase |
|  | 6-phospho-alpha-glucosidase |
|  | arginine/ornithine antiporter |
|  | amino acid ABC transporter ATP-binding protein |
|  | sulfite exporter TauE/SafE family protein |
|  | aluminum resistance protein |
|  | fumarate hydratase |
|  | adherence-associated mucus-binding protein,LPXTG-motif cell wall anchor |
|  | helix-turn-helix domain protein |
|  | tricarballylate dehydrogenase |
|  | amylopullulanase |
|  | alkaline phosphatase |
|  | Pnp/Udp family phosphorylase |
|  | L-2,4-diaminobutyrate decarboxylase |
|  | amidohydrolase |
|  | MATE family efflux transporter |
|  | pyridoxamine 5'-phosphate oxidase |
|  | peptidase propeptide and YPEB domain protein |
|  | phosphotransferase System HPr-Related protein |
|  | short-chain dehydrogenase |
|  | uracil DNA glycosylase superfamily protein |
|  | MarR family transcriptional regulator |
|  | O-acetylhomoserine aminocarboxypropyltransferase |
|  | phage Mu protein F like protein |
|  | homoserine O-succinyltransferase |
|  | dipeptidase PepV |
|  | bacterial SH3 domain protein |
|  | acetoacetate decarboxylase |
|  | rRNA methyltransferase |
|  | catalase |
|  | cobalt ABC transporter permease protein |
|  | ABC-2 family transporter protein |
|  | CsbD-like protein |
|  | glycopeptide antibiotics resistance protein |
|  | peptidoglycan-binding protein |
|  | ATPase involved in chromosome partitioning |
|  | L-threonine kinase |
|  | permease protein |
|  | Thiosulfate sulfurtransferase YnjE precursor |
|  | exopolysaccharide biosynthesis protein |
|  | prolyl-tRNA synthetase |
|  | Xylan alpha-(1->2)-glucuronosidase |
|  | DegV family protein |
|  | YdfK protein |
|  | tagatose-6-phosphate ketose isomerase |
|  | LysR family transcriptional regulator |
|  | 5'(3')-deoxyribonucleotidase |

Table S3. Annotation of genes in generalist and specialist group orthologs. The table indicates the production of genes in each group orthologs and these annotations were based on the genome data from DFAST Archive of Genome Annotation.
