# Supplementary material for: A sugar utilization phenotype contributes to the formation of genetic exchange communities in lactic acid bacteria
Source: FEMS Microbiol Lett. 2021 Sep 1;368(17):fnab117. doi: 10.1093/femsle/fnab117 (PMC8440127; doi:10.1093/femsle/fnab117)
Supplement: fnab117_Supplemental_Files [file fnab117_supplemental_files.zip › Supplementary_data_Figure_S1_minor_revision.docx]

Figure S1(a)

XRE family transcriptional regulator


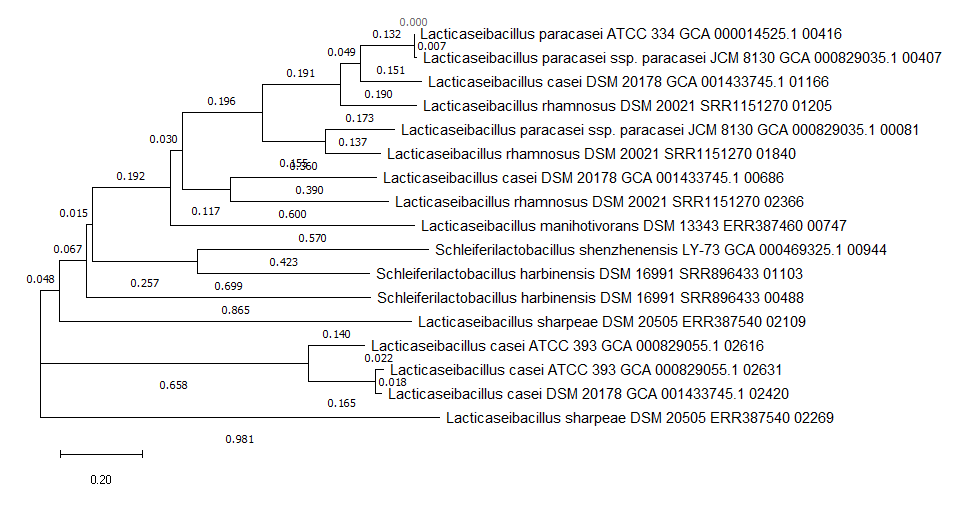


Figure S1(b)

Integral membrane protein PlnU


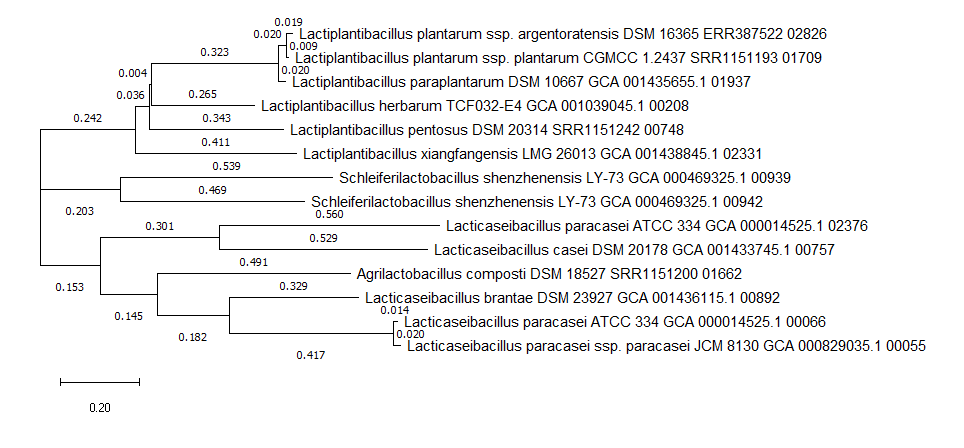


Figure S1(c)

MerR family transcriptional regulator


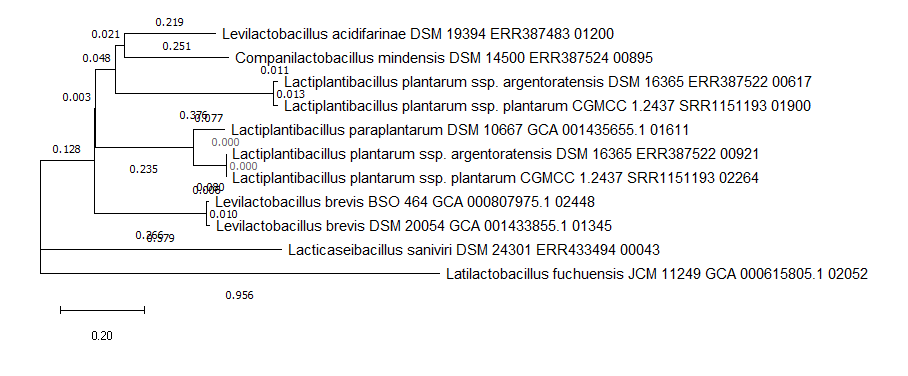


Figure S1(d)

L-fucose isomerase


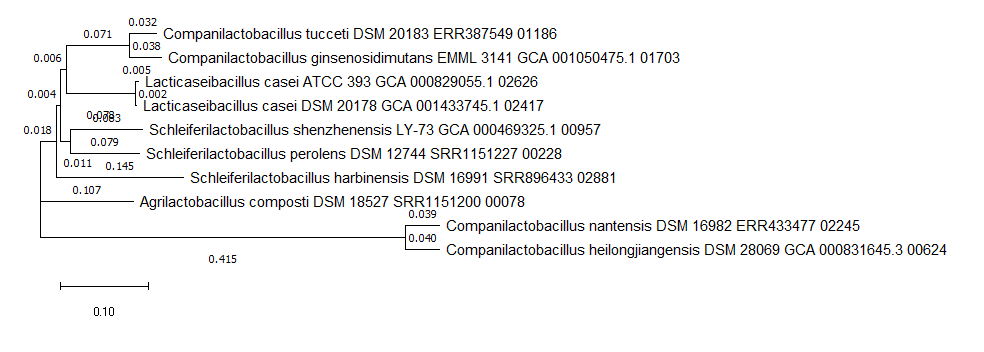


Figure S1(e)

MarR family transcriptional regulator


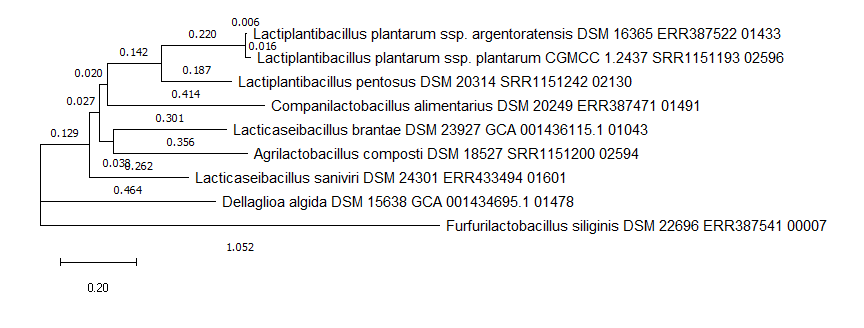


Figure S1. Conflicted phylogenetic trees with the original lineage for the generalist group orthologs. Scale bars are amino acid substitutions per position. In the tree of the xenobiotic response element (XRE) family transcriptional regulator (a), the clade of genus *Lacticaseibacillus* included genes derived from genus *Schleiferilactobacillus*. For the integral membrane protein PlnU (b), although genus *Agrilactobacillus* is closely related to genus *Schleiferilactobacillus* in the tree based on the 16S rRNA gene, the gene derived from *Agrilactobacillus* *composti* were included in the clade for genus *Lacticaseibacillus*. For the mercuric resistance operon regulatory protein (MerR) family transcriptional regulator (c), the gene derived from *Levilactobacillus* *acidifarinae* was distant from the genes of *Levilactobacillus* *brevis* but was included in a clade with a gene for *Companilactobacillus* *mindensis*. For L-fucose isomerase (d), the clade for *Companilactobacillus* *tucceti* and *C*. *ginsenosidimutans* was included in the cluster composed of the genus *Lacticaseibacillus* and *Schleiferilactobacillus* instead of the cluster for *C. nantensis* and *C. heilongjiangensis*. For the multiple antibiotic resistance protein (MarR) family transcriptional regulator (e), the gene of *Lacticaseibacillus* *brantae* formed a clade with the gene of *Agrilactobacillus composti* instead of the gene derived from *Lacticaseibacillus sanviri*.
