# Supplementary material for: A sugar utilization phenotype contributes to the formation of genetic exchange communities in lactic acid bacteria
Source: FEMS Microbiol Lett. 2021 Sep 1;368(17):fnab117. doi: 10.1093/femsle/fnab117 (PMC8440127; doi:10.1093/femsle/fnab117)
Supplement: fnab117_Supplemental_Files [file fnab117_supplemental_files.zip › Supplementary_data_legends_minor_revision.docx]

**Supplementary data**

Figure S1. Conflicted phylogenetic trees with the original lineage for the generalist group orthologs. Scale bars are amino acid substitutions per position. In the tree of the xenobiotic response element (XRE) family transcriptional regulator, the clade of genus *Lacticaseibacillus* included genes derived from genus *Schleiferilactobacillus*. For the integral membrane protein PlnU, although genus *Agrilactobacillus* is closely related to genus *Schleiferilactobacillus* in the tree based on the 16S rRNA gene, the gene derived from *Agrilactobacillus* *composti* were included in the clade for genus *Lacticaseibacillus*. For the mercuric resistance operon regulatory protein (MerR) family transcriptional regulator, the gene derived from *Levilactobacillus* *acidifarinae* was distant from the genes of *Levilactobacillus* *brevis* but was included in a clade with a gene for *Companilactobacillus* *mindensis*. For L-fucose isomerase, the clade for *Companilactobacillus* *tucceti* and *C*. *ginsenosidimutans* was included in the cluster composed of the genus *Lacticaseibacillus* and *Schleiferilactobacillus* instead of the cluster for *C. nantensis* and *C. heilongjiangensis*. For the multiple antibiotic resistance protein (MarR) family transcriptional regulator, the gene of *Lacticaseibacillus* *brantae* formed a clade with the gene of *Agrilactobacillus composti* instead of the gene derived from *Lacticaseibacillus sanviri*.

Table S1. Features of the 178 LAB strains. The accession numbers of the genome sequences, the old and new species names, strain names, type status, seven genomic features, six phenotypic characteristics, and the strains’ isolation source are presented. The genomic features are genome size (bp), number of CDS, G/C content (%), number of rRNA, number of tRNA, number of CRISPRs, number of CDS judged to be HGTs. One of phenotypes is sugar utilization value which indicates the number of sugar types that can be utilized. The other five phenotypes, growth at 15 °C, growth at 45 °C, and growth in microaerobic, facultatively anaerobic, and obligate anaerobic conditions were expressed as a dummy variable: If a strain has the feature, 1 was given as the dummy variable and 0 if not. The isolation source indicates the environment in which the species was isolated.

Table S2. Community extraction of the networks of the shared generalist group orthologs. The table indicates the number of strains, genera name, and member in each community for the generalist group ortholog networks.

Table S3. Annotation of genes in generalist and specialist group orthologs. The table indicates the production of genes in each group orthologs and these annotations were based on the genome data from DFAST Archive of Genome Annotation.
